# Influenza A Virus Polymerase Recruits the RNA Helicase DDX19 to Promote the Nuclear Export of Viral mRNAs

**DOI:** 10.1038/srep33763

**Published:** 2016-09-22

**Authors:** Cédric Diot, Guillaume Fournier, Mélanie Dos Santos, Julie Magnus, Anastasia Komarova, Sylvie van der Werf, Sandie Munier, Nadia Naffakh

**Affiliations:** 1 Institut Pasteur, Unité de Génétique Moléculaire des Virus à ARN, Département de Virologie, F-75015 Paris, France; 2 CNRS, UMR3569, F-75015 Paris, France; 3 Université Paris Diderot, Sorbonne Paris Cité, Unité de Génétique Moléculaire des Virus à ARN, EA302, F-75015 Paris, France; 4 Institut Pasteur, Unité de Génomique Virale et Vaccination, Département de Virologie, F-75015 Paris, France

## Abstract

Enhancing the knowledge of host factors that are required for efficient influenza A virus (IAV) replication is essential to address questions related to pathogenicity and to identify targets for antiviral drug development. Here we focused on the interplay between IAV and DExD-box RNA helicases (DDX), which play a key role in cellular RNA metabolism by remodeling RNA-RNA or RNA-protein complexes. We performed a targeted RNAi screen on 35 human DDX proteins to identify those involved in IAV life cycle. DDX19 was a major hit. In DDX19-depleted cells the accumulation of viral RNAs and proteins was delayed, and the production of infectious IAV particles was strongly reduced. We show that DDX19 associates with intronless, unspliced and spliced IAV mRNAs and promotes their nuclear export. In addition, we demonstrate an RNA-independent association between DDX19 and the viral polymerase, that is modulated by the ATPase activity of DDX19. Our results provide a model in which DDX19 is recruited to viral mRNAs in the nucleus of infected cells to enhance their nuclear export. Information gained from this virus-host interaction improves the understanding of both the IAV replication cycle and the cellular function of DDX19.

The DExD-box RNA (DDX) helicases form the largest family within the helicases superfamily 2 (SF2)^1^. They share the ability to remodel RNA-RNA or RNA-protein complexes in an ATP dependent manner and they play major roles in all aspects of the cellular RNA metabolism^2^. Most DDX helicases contain 9 canonical sequence motifs that are involved in ATP hydrolysis, ATP binding and RNA binding. Structurally, they share a common core composed of 2 RecA-like domains forming a cleft lined by the conserved sequence motifs^3^.

RNA viruses have relatively small genomes that encode a limited number of proteins, and have evolved so that they hijack cellular components and cellular pathways to facilitate their replication. In particular, a growing list of RNA viruses were found to co-opt DDX proteins to support various steps of their life cycle. For instance, the HIV-1 Rev protein associates with DDX3, which together with DDX1 promotes the Rev-dependent nuclear export of unspliced and singly-spliced viral mRNAs^4^. Although the HCV genome encodes a viral RNA helicase, DDX3, DDX1 and DDX6 are required for efficient HCV genomic RNA replication^5^. The DDX1 protein was shown to interact with the Nsp14 exonuclease of coronaviruses and to facilitate their replication^6^. The DDX proteins may also be involved at later stages of viral infection, as exemplified by the role of DDX24 in the packaging of HIV-1 RNA during virus assembly^7^ or the role of DDX56 in the assembly of West Nile virus particles^8^. In addition several DDX helicases have been involved in anti-viral innate immunity, mostly as sensors. This is notably the case for DDX58, also named RIG-I, but also for DDX3, DDX41, DDX1, DDX21 and DDX60^9^.

The genome of influenza A viruses (IAV) does not encode any recognized RNA helicase. It consists of eight single-stranded RNA segments of negative polarity (vRNAs), each segment being encapsidated with the nucleoprotein (NP) and associated with the viral RNA-dependent RNA polymerase to form viral ribonucleoproteins (vRNPs). Upon viral entry by endocytosis, the incoming vRNPs are released in the cytoplasm and imported in the nucleus. The viral heterotrimeric polymerase, formed by the PB1, PB2 and PA subunits, ensures the transcription of vRNAs into mRNAs, and their replication via the synthesis of full-length complementary RNAs (cRNAs) which then serve as templates for the synthesis of vRNAs^10^. Viral mRNAs are capped as a result of a “cap-snatching” mechanism of transcription priming, and polyadenylated through the stuttering of the viral polymerase at a stretch of five to seven U residues close to the 5′ end of the vRNA template. Most of the viral mRNAs are intronless except for the M1, NS1, and PB2 mRNAs that can undergo splicing^10^,^11^. The mechanisms by which viral mRNAs are exported to the cytoplasm to be translated remain largely unknown.

The list of cellular proteins that can bind to the components of vRNPs and/or play a role in viral replication keeps expanding^12^,^13^. However, the interplay between DDX helicases and IAV is still scarcely documented. DDX21 was recently found to interact sequentially with the viral proteins PB1 and NS1, and to contribute to the temporal regulation of viral genes expression^14^. DDX39B, also named UAP56/BAT1, enhances viral RNA synthesis by the viral polymerase^15^, prevents the formation of double-stranded RNA^16^, and promotes the nuclear export of the viral M1 and M2 mRNAs^17^,^18^. DDX17 seems to enhance or to reduce the viral polymerase activity depending on the human or avian origin of the virus^19^. Genome-wide RNAi screens have pointed to other DDX proteins such as DDX2B, DDX3X, DDX5 and DDX55 as being potentially involved in the IAV life cycle^12^,^13^,^19^.

Here we specifically investigated the importance of a selected set of 35 human DDX helicases in IAV replication by performing a targeted siRNA screen. Fourteen DDX proteins were found to contribute to viral multiplication. Among these, DDX19 appeared to be strongly required for influenza virus replication, and we therefore characterized its role in the IAV life cycle. DDX19 (DBP5 in yeast) is an evolutionary conserved factor in eukaryotes, which shuttles between the nucleus and the cytoplasm and is associated to the cytoplasmic face of the nuclear pore complexes (NPCs)^20^,^21^,^22^. It plays a major role in promoting the directional export of cellular messenger ribonucleoprotein particles (mRNPs) through the NPCs^3^,^20^,^23^. Recent studies revealed that the function of DDX19 in nucleo-cytoplasmic trafficking is not restricted to mRNPs, as it was found to mediate the nuclear export of pre-ribosomal subunits^24^ and the nuclear import of the MLK1 transcription factor in an RNA-dependent manner^25^. In addition, DDX19 appears to control the fate of cellular RNAs at multiple levels from transcription to translation^26^.

Different mechanisms of action have been proposed, all of which involve sequential binding of DDX19 to two cytoplasmic nucleoporins, GLE1 and NUP214, to ensure coupling of the DDX19 ATPase cycle to mRNP remodeling and nuclear export^27^,^28^. These models are based on biochemical and structural data obtained mainly *in vitro* on the isolated yeast homologs DBP5, GLE1 and NUP159. The function and mRNA targets of DDX19 in human cells have been little documented so far, and the mechanism of its recruitment to mRNPs remains unclear. Here we show that DDX19 associates with intronless, unspliced and spliced IAV mRNAs in infected cells and is involved in their nuclear export. In addition we give evidence for an association between the viral polymerase and DDX19, which is not mediated by RNA, and is modulated by the ATPase activity of DDX19. Altogether, our data provide a model in which DDX19 is recruited by viral mRNPs in the nucleus of infected cells to enhance their nuclear export.

## Results

### RNAi screening of DDX RNA helicases points to DDX19 as a factor required for efficient IAV multiplication

A targeted RNAi screen was performed to identify human DDX proteins involved in IAV multiplication. Small interfering RNAs targeting 35 DDX proteins (Table S1) were transfected in A549 cells. For DDX2A/DDX2B, DDX19A/DDX19B and DDX39A/DDX39B, siRNAs targeting the A and B forms were used alone or in combination. Anti-Nup62 siRNAs, known to inhibit IAV replication^29^,^30^, were used as a control. The toxicity of siRNA treatment and the silencing efficiency were evaluated as described in the Methods section. No reduction of the cell viability signal was measured compared to control siRNA-treated cells, except in cells silenced for DDX2A, DDX2A + B, DDX39A + B and DDX48 (Figure S1a). Efficient silencing (>70% decrease of the protein expression signal) was achieved for most DDX proteins except for DDX2B, 13, 18, 20, 31 and 52 (16% to 69% decrease) (Figure S1b).

**Figure 1:**
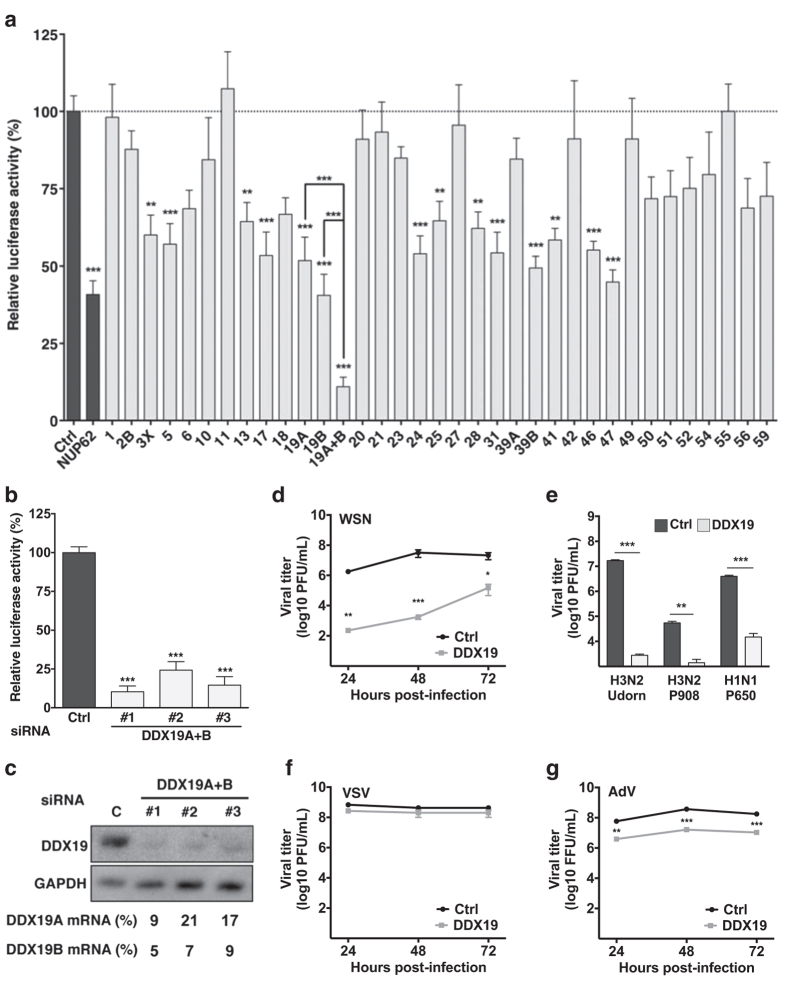
IAV multiplication is reduced in DDX19-depleted cells. (**a,b**) A549 cells were treated with control non-target or NUP62 siRNAs (dark grey bars) or siRNAs targeting the indicated DDX (light grey bars) and infected with the WSN-PB2-Nanoluc virus (0.0001 pfu/cell). Luciferase activities were measured in cell lysates prepared at 24 hpi. Three independent experiments were performed in triplicate. The results are expressed as the mean percentages ± SEM of luciferase activity relative to the non-target siRNA condition, and the significance was tested with a Holm-Sidak’s multiple comparisons test using GraphPad Prism software (**p < 0.01; ***p < 0.001). (**c**) At 48 hours post-transfection (hpt) with the control non-target siRNAs (C) or with individual siRNAs targeting DDX19A+B (#1, #2 and #3), the levels of DDX19 protein were evaluated by immunoblot using an antibody that recognizes both A and B forms of DDX19, and the levels of DDX19A and DDX19B mRNAs were determined by RT-qPCR and were expressed as percentages of the control. Cropped blots are shown. The corresponding full-length blots are shown in Figure S2. (**d**–**g**) A549 cells were treated with control (dark grey lines) or DDX19 siRNAs (light grey lines) and infected with the following viruses at the indicated m.o.i. in pfu/cell: A/WSN/33(H1N1 WSN, 0.0001); A/Udorn/307/72(H3N2) (Udorn, 0.01); A/Paris/908/97(H3N2) (P908, 0.01); A/Paris/650/2004(H1N1) (P650, 0.01); Vesicular Stomatitis Virus (VSV, 0.0001); Adenovirus 5 (AdV, 6). At the indicated times post-infection (**d,f,g**) or at 24 hpi (**e**), the viral titers were determined by plaque assay on MDCK cells (**d**–**f**) or immunostaining (**g**). The results are expressed as the mean ± SEM of triplicates and the significance was tested with an unpaired, 2-tailed Student t test using GraphPad Prism software (*p < 0.05; **p < 0.01; ***p < 0.001).

A549 cells were transfected with each of the non-toxic siRNAs and subsequently infected at a low multiplicity of infection (m.o.i.) with a recombinant A/WSN/33 virus carrying a luciferase reporter gene (WSN-PB2-Nanoluc). Luciferase activity was measured in cell lysates prepared at 24 hours post-infection (hpi) to monitor the efficiency of viral replication. As shown in Fig. 1a, IAV replication was significantly impaired upon silencing of 14 DDX proteins: DDX3X, DDX5, DDX13, DDX17, DDX19A, DDX19B, DDX24, DDX25, DDX28, DDX31, DDX39B, DDX41, DDX46 and DDX47. Among the DDX proteins that had been previously found to positively regulate IAV replication, DDX3X, DDX5 and DDX17^19^ and DDX39B^15^ were recovered in our screen, but not DDX2B, possibly due to low knock-down efficiency (Figure S1b). DDX21 silencing, shown by others to negatively regulate IAV replication^14^, had no significant effect in our screen. Upon co-silencing of DDX19 A and B forms (96% identity at the protein level), the reduction of luciferase signal was significantly greater than upon silencing of DDX19A or DDX19B alone (a 90% reduction compared to 48% and 59%, respectively, Fig. 1a), indicating a synergistic effect of DDX19A and DDX19B depletion. We repeated the experiment with three individual siRNAs targeting conserved regions between DDX19A and DDX19B (Fig. 1b,c). A strong 76 to 90% reduction of the luciferase signal was observed with all three individual siRNAs (Fig. 1b), thus ruling out any off-target effect. In the experiments described below, the combination of DDX19A and DDX19B siRNAs (thereafter named DDX19 siRNAs) was used.

The effect of DDX19 knock-down on the multiplication of several wild-type IAV was then examined. A549 cells treated with DDX19 or control siRNAs were infected at low m.o.i. and the production of infectious viral particles was evaluated at 24, 48 and 72 hpi for A/WSN/33 (WSN, Fig. 1d), and at 24 hpi for the other strains (Fig. 1e). At all time points, the WSN virus titers in the supernatant of DDX19-silenced cells were decreased by 2–4 log compared to control cells (Fig. 1d). At 24 hpi, similar reductions were observed upon infection with the A/Udorn/307/72(H3N2), A/Paris/908/97(H3N2) or A/Paris/650/2004(H1N1) viruses (Fig. 1e). No reduction was observed with the VSV Indiana strain (Fig. 1f), a negative-stranded RNA virus from the *Rhabdoviridae* family that replicates in the cytoplasm. In contrast, the production of infectious Adenovirus 5, a double-stranded DNA virus from the *Adenoviridae* family, was decreased by 1-log upon DDX19 depletion (Fig. 1g). These results confirm that DDX19 is essential for IAV multiplication, and suggest its potential implication in the life cycle of other viruses that replicate in the nucleus.

### DDX19 silencing reduces the accumulation of viral proteins and RNAs

**Figure 2:**
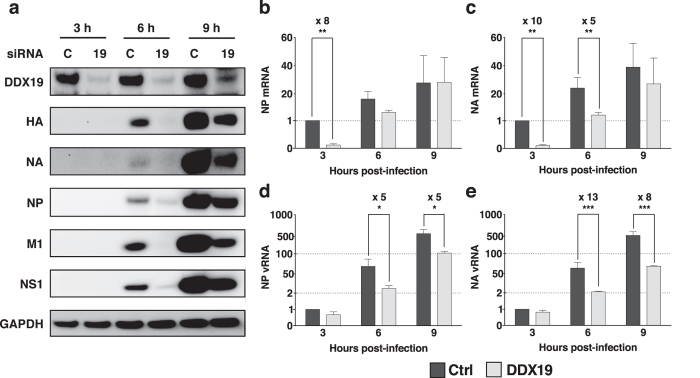
The accumulation of viral proteins and RNAs is reduced in DDX19-depleted cells. A549 cells were treated with control (C) or DDX19 (19) siRNAs and infected with WSN (5 pfu/cell). (**a**) Total extracts were prepared at the indicated times post-infection and analyzed by immunoblots using antibodies directed against the indicated proteins. Results representative of 3 independent experiments are shown. Cropped blots are shown. The corresponding full-length blots are shown in Figure S3. (**b**–**e**) The levels of NP or NA mRNAs and vRNAs (**b** or **c** and **d** or **e**, respectively) were determined at the indicated times post-infection by strand specific RT-qPCR and were normalized to the level of the same RNA species at 3 hpi in cells treated with the control siRNAs. The results are expressed as the mean ± SEM of three independent experiments and the significance was tested with a one-sample t test using GraphPad Prism Software (*p < 0.05; **p < 0.01; ***p < 0.001). Dashed lines were used to indicate that the Y-axes have been segmented. Different scales were used for the mRNA and vRNA graphs.

To further document the role of DDX19 in IAV multiplication, the temporal accumulation of viral components was analyzed in DDX19-silenced cells during single-cycle infection with WSN. As shown in Fig. 2a, in control A549 cells, the accumulation of HA, NA, NP, M1 and NS1 viral proteins was detectable from 6 hpi. In DDX19-silenced cells, the accumulation of viral proteins was no longer (HA, NA, M1) or weakly (NP, NS1) detectable at 6 hpi, and was strongly reduced at 9 hpi compared to control cells.

Monitoring of NP and NA vRNAs and mRNAs using strand-specific RT-qPCR^31^ showed that mRNA levels were reduced at 3 hpi in DDX19-silenced cells compared to control cells (8-fold for NP and 10-fold for NA on average), while no significant difference was observed at later time points, except for NA mRNAs at 6 hpi (Fig. 2b,c). The effect on the accumulation of NP and NA vRNAs was delayed, as vRNA levels were strongly reduced at 6 hpi and still so at 9 hpi (5- and 5-fold for NP and 13- and 8-fold for NA at 6 and 9 hpi, respectively) (Fig. 2d,e). We hypothesize that mRNA levels appear less affected at 6 and 9 hpi because their accumulation has plateaued at these time points, unlike the viral vRNAs which continue to accumulate at a high rate. Notably, for each of the vRNA and mRNA species that were examined, the levels measured in DDX19-depleted cells at 6 and 9 hpi were similar to the levels measured in control cells at 3 and 6 hpi, respectively. Taken together, these data indicate that DDX19 silencing does not distinctly affect the process of transcription/replication *per se* but delays the accumulation of viral RNAs from early time points of infection, which disrupts the whole viral cycle.

### Nuclear import of parental vRNAs and primary transcription are not affected by DDX19 silencing

**Figure 3:**
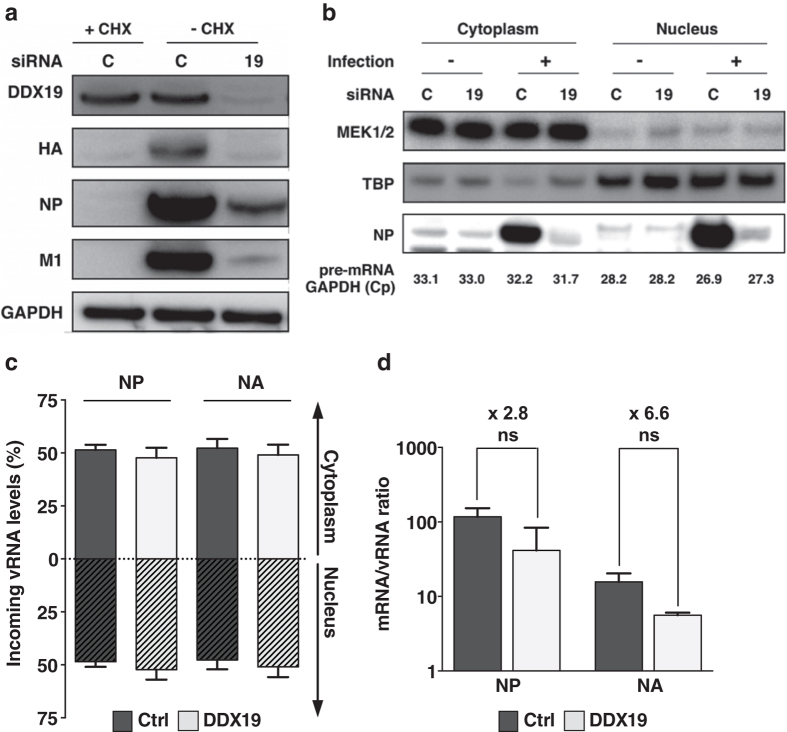
The earliest steps of IAV replication are not affected by DDX19 depletion. A549 cells treated with control (C in a and b, dark grey bars in c and d) or DDX19 (19 in a and b, light grey bars in c and d) siRNAs were infected with WSN (5 pfu/cell). (**a**) Total extracts from control cells treated with CHX (+CHX) or not (−CHX) were prepared at 6 hpi and analyzed by immunoblots using antibodies directed against the indicated proteins. Cropped blots are shown. The corresponding full-length blots are shown in Figure S4. (**b**) Cytoplasmic and nuclear fractions were prepared at 4 hpi. Aliquots of the indicated subcellular fractions were analyzed by immunoblots with antibodies directed against MEK1/2 kinase (cytoplasmic marker), TBP (nuclear marker) and NP. Alternatively, total RNAs were extracted and the levels of GAPDH pre-mRNA, a nuclear marker, were determined by real-time RT-PCR. Results are expressed as the mean of two determinations of the crossing point value (Cp). Cropped blots are shown. The corresponding full-length blots are shown in Figure S5. (**c,d**) Infection was carried out for 6 h in the presence of CHX (100 µg/mL). Total RNAs were isolated from cytoplasmic (solid bars) and nuclear (hatched bars) fractions, and the levels of NP and NA vRNAs were determined by strand specific RT-qPCR. The results are expressed as the mean percentages ± SEM of cytoplasmic and nuclear vRNAs levels determined in three independent experiments (c). Total RNAs were extracted and the levels of NP or NA mRNAs and vRNAs were determined by strand specific RT-qPCR. The results are expressed as the mean ratios of mRNA/vRNA ± SEM determined in three independent experiments (d).

To test whether the nuclear import of parental vRNAs is affected by DDX19 silencing, A549 cells were treated with cycloheximide (CHX) during infection to inhibit *de novo* viral protein expression, and incoming vRNAs were monitored in subcellular fractions. As shown in Fig. 3a, no viral protein accumulation was detected at 6 hpi in the presence of CHX, demonstrating the efficiency of CHX treatment. The quality of subcellular fractionation was controlled in each experiment by immunoblotting using antibodies against the MEK1/2 kinase, a cytoplasmic marker, and the TATA Binding Protein (TBP), a nuclear marker. In addition, the levels of GAPDH pre-mRNAs were evaluated by real-time RT-PCR and used as a nuclear marker. Representative data are shown in Fig. 3b. The quality of subcellular fractionation was not affected by viral infection (Fig. 3b).

Strand-specific RT-qPCR was used to monitor the levels of NP and NA vRNAs in the cytoplasmic and nuclear fractions of CHX-treated cells, as well as the level of primary transcription from these incoming vRNA templates. The proportion of nuclear vRNAs (about 50%) was similar in DDX19-silenced or control cells (Fig. 3c), thus indicating that the nuclear import of parental vRNAs was not impaired. As shown in Fig. 3d, mRNA/vRNA ratios were reduced after DDX19 silencing in CHX-treated cells. However the differences were not statistically significant, indicating that DDX19 has, if any, a limited effect on primary transcription. Altogether, these results demonstrate that DDX19 does not have a critical function in the earliest steps of the influenza viral cycle.

### DDX19 contributes to the nuclear export of viral transcripts

**Figure 4:**
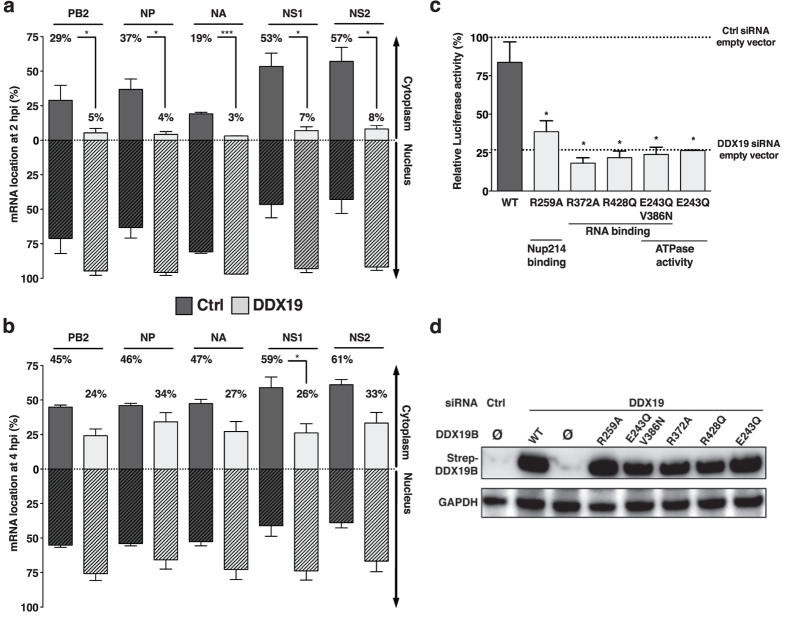
The nuclear export of IAV mRNAs is dependent on DDX19. (**a**,**b**) A549 cells were treated with control (dark grey bars) or DDX19 (light grey bars) siRNAs and infected with WSN (5 pfu/cell). Cytoplasmic and nuclear extracts were prepared at 2 hpi (**a**) or 4 hpi (**b**), and poly(A)+ mRNAs were purified. The levels of viral mRNAs were determined by RT-qPCR. The percentages of mRNAs detected in the cytoplasmic fraction (solid bars) and nuclear fraction (hatched bars) are shown. The results are expressed as the mean ± SEM of three independent experiments, and the significance was tested with paired t test using GraphPad Prism software (*p < 0.05; ***p < 0.001). (**c**,**d**) HEK-293T cells were transfected with DDX19 or control siRNAs and then with siRNA-resistant DDX19B plasmids or empty vector. After 24 h, cells were infected with the WSN-PB2-Nanoluc virus (0.01 pfu/cell) and luciferase activities were measured at 24 hpi (**c**). The results are expressed as the mean percentages ± SEM of luciferase activity relative to the control siRNA + empty vector condition, and the significance was tested with a Holm-Sidak’s multiple comparisons test using GraphPad Prism software (*p < 0.05). (**d**) Lysates from the RNAi rescue experiment shown in c were analyzed by immunoblot using Strep-Tactin or anti-GAPDH antibodies. The ⦸, symbol indicates cells transfected with the empty vector. Cropped blots are shown. The corresponding full-length blots are shown in Figure S6.

We next examined the potential role of DDX19 in the export of viral mRNAs. To this end, siRNA-treated A549 cells were infected and subjected to subcellular fractionation at 2 and 4 hpi. The levels of cytoplasmic and nuclear PB2, NP, NA, NS1 and NS2 mRNAs were then determined by RT-qPCR (Fig. 4a,b). At 2 hpi in control cells, a substantial proportion of each mRNA species (29% to 57%) was found in the cytoplasm (Fig. 4a, dark grey solid bars). In DDX19-silenced cells, the proportions of cytoplasmic viral mRNAs were strikingly reduced (3% to 8%) (Fig. 4a, light grey solid bars). The sensitivity of the M1- and M2-specific RT-qPCR was insufficient to evaluate accurately the levels of cytoplasmic and nuclear M1 and M2 mRNAs at 2 hpi. At 4 hpi in control cells, the viral mRNAs appeared equally distributed in both fractions (PB2, NP, NA) or predominantly cytoplasmic for NS1 and NS2 mRNAs (Fig. 4b). In contrast, in DDX19-silenced cells, the viral mRNAs remained predominantly nuclear, with only 24% to 34% of the viral mRNAs being detected in the cytoplasm (Fig. 4b). Although these differences were repeatedly observed (n = 3), the effect of DDX19 silencing at 4 hpi was generally not significant upon statistical analysis.

The function of the homolog of DDX19 in yeast has been extensively studied and mutants defective for RNA binding (R372G, V386N, R428Q) or ATPase activity (E243Q)^20^,^32^, or NUP214 binding (R259A)^33^,^34^ show strong defects in the nuclear export of mRNAs. To confirm that these activities contribute to DDX19 function in IAV replication, we performed RNAi rescue experiments (Fig. 4c). Upon silencing of DDX19 in HEK-293T cells, siRNA-resistant expression plasmids for the wild-type or mutant DDX19B proteins were transfected. Expression levels of the different DDX19B mutant proteins did not differ significantly (Fig. 4d), and published data indicate that these are properly folded^25^,^34^. The efficiency of viral replication was then assessed using the WSN-PB2-Nanoluc virus (Fig. 4c). The wild-type DDX19B plasmid (dark grey bar) could rescue >75% of the level of viral replication. This apparent contradiction with the fact that upon depletion, both DDX19A and DDX19B appear to play a role in viral infection (Fig. 1a), is likely due to a high expression level of the recombinant DDX19B protein. Unlike the wild-type DDX19B plasmid (dark grey bar), none of the mutant plasmids tested (R259A, R372A, R428Q, E243Q, and E243Q/V386N, light grey bars) rescued the level of viral replication. Thus all activities related to DDX19B function in nuclear export of mRNAs appear to be required for IAV replication. Taken together with the nuclear retention of viral mRNAs in DDX19 depleted cells, our data clearly establish the essential contribution of DDX19 to the nuclear export of IAV mRNAs.

### DDX19 binds the viral polymerase and viral RNAs during infection

We previously reported the interaction between DDX19A/B and the viral polymerase in an infectious context, using a Gaussia luciferase complementation assay named iPCA (infectious Protein Complementation Assay)^29^. In this system, a recombinant WSN virus that encodes a Gluc1-tagged polymerase subunit is used to infect cells transiently expressing a protein of interest fused to Gluc2; a significant luciferase activity is restored only if Gluc1 and Gluc2 are brought in close proximity by interacting proteins^29^.

**Figure 5:**
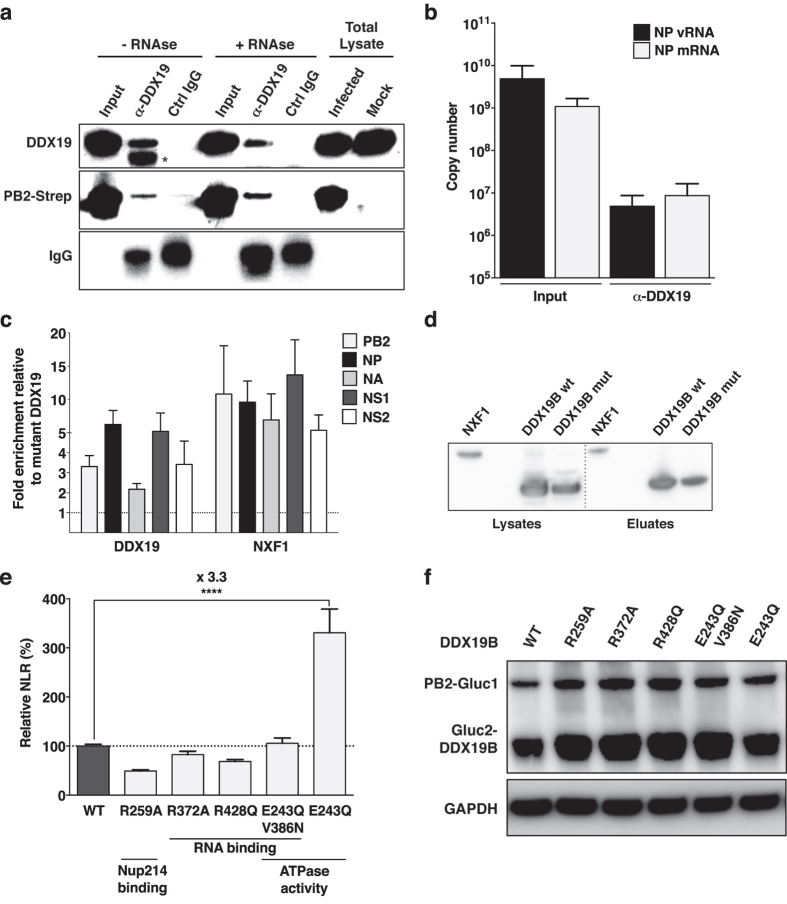
The viral polymerase and viral RNAs interact with DDX19. (**a**,**b**) A549 cells were infected with WSN (5 pfu/cell). At 3.5 hpi, DDX19 proteins were purified in the absence or presence of RNAse, using anti-DDX19 antibodies (α-DDX19) or control immunoglobulins (Ctrl IgG). (**a**) Inputs and α-DDX19/Ctrl IgG eluates were analyzed by immunoblot to detect DDX19, PB2-Strep and the IgG antibodies (upper, middle and lower panel, respectively). Results representative of two independent experiments are shown. The star indicates a non-specific band. (**b**) The levels of NP mRNAs and vRNAs co-purified with DDX19 are expressed as the mean ± SEM of four independent experiments. The background level of detection in control IgG eluates was 2.9 × 10^6^ and 3.9 × 10^5^ copies for NP vRNAs and NP mRNAs, respectively. Co-immunoprecipitated to input copy ratios between NP mRNAs and vRNAs differed significantly (multivariate linear model with an interaction term between sample type and RNA type, p = 0.0465). (**c**,**d**) HEK-293T cells expressing Strep-tagged wild-type DDX19B, mutant DDX19B or NXF1 were infected with WSN (5 pfu/cell). At 6 hpi, Strep-tagged proteins were purified, and the levels of co-purified mRNAs were determined (**c**). For wild-type DDX19B and NXF1, co-purified/total mRNAs ratios were normalized to the one obtained with the DDX19 mutant. The data represent the mean ± SEM of three independent experiments. Statistical analysis (multivariate linear model) did not demonstrate significance. (**d**) Lysates and eluates were analyzed by immunoblot using Strep-Tactin. The dashed line indicates the juxtaposition of non-adjacent lanes from the same immunoblot. (**e**,**f**) HEK-293T cells expressing the Gluc2-DDX19B variants were infected with the WSN-PB2-Gluc1 virus at a m.o.i. >1 pfu/cell. At 6 hpi, cells were lysed and normalized luminescence ratios (NLRs) were determined (**e**). Four independent experiments in triplicate were performed. The results are expressed as the mean percentages ± SEM of luciferase activity relative to the DDX19B wild-type condition. The significance was tested with a Holm-Sidak’s multiple comparisons test using GraphPad Prism software (****p < 0.0001). (**f**) Lysates were analyzed by immunoblot using anti-Gluc or anti-GAPDH antibodies. Cropped blots are shown in (**a**,**d**,**f**). Corresponding full-length blots are shown in Figures S7, S8 and S9, respectively.

To confirm the interaction of the endogenous DDX19 protein with the viral polymerase during infection and to assess whether this interaction requires the presence of RNAs, DDX19 was immunoprecipitated from lysates of HEK-293T cells infected with a recombinant WSN virus expressing a Strep-tagged PB2 protein (WSN-PB2-Strep) in the presence or absence of RNAse (Fig. 5a). Upon DDX19 immunoprecipitation (Fig. 5a, upper panel), the PB2-Strep protein was specifically co-immunoprecipitated in conditions where RNA integrity was preserved (Fig. 5a, middle panel, -RNase) or not (Fig. 5a, middle panel, +RNase). RNAs from cell lysates and eluates were extracted and the viral NP mRNAs and vRNAs were quantified by strand-specific RT-qPCR. They could not be detected in RNase-treated samples (data not shown), thus demonstrating the efficiency of RNase treatment. In RNase-free samples, a higher proportion of NP mRNAs was co-immunoprecipitated with DDX19 (8.6 × 10^6^ copies −0.79% of the input NP mRNAs) compared to NP vRNAs (4.8 × 10^6^ copies −0.10% of the input NP vRNAs) (p = 0.0465) (Fig. 5b).

To confirm the specificity of viral mRNAs association with DDX19, we determined how efficiently they were co-purified with a recombinant Strep-tagged wild-type DDX19B protein over its RNA-binding and ATPase defective mutant counterpart (the R372A-V386N-R428Q and E243Q mutant) (Fig. 5c). A Strep-tagged NXF1 protein was used as a positive control, as NXF1 was shown to bind IAV mRNAs^30^,^35^. Upon transient expression in HEK-293T cells and subsequent infection with the WSN virus, the Strep-tagged proteins were purified (Fig. 5d), and the PB2, NP, NA, NS1 and NS2 mRNAs were quantified by RT-qPCR on 1 μg of RNA extracted from the cell lysate (C) and eluate (E) fractions. The E/C ratios of mRNAs levels were determined for each protein and normalized to those obtained with the DDX19B mutant protein. As shown in Fig. 5c, the PB2, NP, NA, NS1 and NS2 mRNAs were enriched 2- to 6-fold and 5- to 13-fold upon co-purification with the Strep-DDX19B and -NXF1 protein, respectively, compared to the DDX19 mutant protein.

To further dissect the connection between DDX19-RNA and DDX19-polymerase interactions, we used iPCA to assess the capacity of mutant DDX19 proteins to interact with PB2 (Fig. 5e). HEK-293T cells transiently expressing the wild-type or mutant DDX19 proteins were subsequently infected with the WSN-PB2-Gluc1 virus. All DDX19 mutants fused to Gluc2 were expressed at similar levels (Fig. 5f). As shown in Fig. 5e, similar DDX19-PB2 interaction signals were observed with DDX19 mutants defective for RNA binding and with the wild-type protein. These results are in agreement with our co-immunoprecipitation data showing that the DDX19-polymerase interaction is RNA-independent (Fig. 5a). Interestingly, in the presence of the E243Q mutant, a significant increase of the DDX19-PB2 interaction signal was observed compared to the wild-type or to the E243Q-V386N double-mutant DDX19 protein (Fig. 5e). The E243Q mutant was shown to be deficient for ATPase activity but, unlike the E243Q-V386N double-mutant, to retain a strong RNA binding activity^20^. Our data show that these properties result in the stabilization of the DDX19-PB2 interaction. Altogether, our results demonstrate an RNA-independent association between DDX19 and the viral polymerase, that is modulated by the ATPase activity of DDX19.

## Discussion

Here we provide the first targeted RNAi screening of the DExD-box RNA helicases family, which is the largest and most diverse group of human RNA helicases, to identify those required for efficient IAV replication. The hits of our RNAi screen include, in addition to DDX3X, DDX5, DDX17 and DDX39B that had been found previously to promote IAV replication^15^,^19^, 10 new cellular factors potentially involved in IAV replication, *i.e.* DDX13, DDX19A/B, DDX24, DDX25, DDX28, DDX31, DDX41, DDX46 and DDX47. The most dramatic decrease in IAV replication was observed upon silencing of DDX19, which was therefore analyzed in more details. The roles of the other DDX hits remain to be investigated. Given the multi-functional nature of DDX proteins, each of them could potentially be involved at various steps of the viral cycle. Their recognized functions include splicing of pre-mRNAs for DDX41^36^ and DDX46^37^, mRNA export and protein translation for DDX19 and DDX25^26^,^38^, exosome-mediated RNA decay for DDX13, also named SKIV2L^39^, ribosome or mitoribosome biogenesis for DDX47^40^, DDX31^41^ and DDX28^42^, modulation of signaling pathways for DDX31^41^, DDX24^43^ and DDX41^44^.

Our study reveals that the cellular DDX19 protein plays an essential role during IAV infection by mediating viral mRNA export. Assuming that the regulatory mechanisms described for the yeast homolog of DDX19 are conserved in humans, DDX19 has a mRNP remodeling activity which depends on its RNA binding activity as well as its NUP214 binding and ATPase activities^27^,^28^. This is consistent with our observation that DDX19 depletion i) inhibits the nuclear export of IAV mRNAs (Fig. 4a) and ii) leads to a reduction in viral replication that can be rescued by re-expressing the wild-type DDX19 protein, but not any of the mutants defective for RNA binding, NUP214 binding or ATPase activity (Fig. 4c). The effect of DDX19 depletion on the nucleo-cytoplasmic distribution of viral mRNAs was detectable at 2 hpi and to a lesser extent at 4 hpi (Fig. 4a,b), suggesting that the requirement for DDX19 can be bypassed as viral infection proceeds. The accumulation of high levels of viral mRNAs in the nucleus and/or virus-induced modifications of the cellular environment (e.g. altered permeability of the nuclear envelope^45^) could allow viral mRNAs to be exported from the nucleus independently of DDX19. Still, in DDX19-depleted cells the inhibition of viral mRNA export at early time points results in a considerably delayed viral life cycle. By impairing the production of viral RNAs and viral proteins (Fig. 2), it strongly reduces the production of infectious virus particles as observed with several IAV strains (Fig. 1e). A possible role of DDX19 in the life cycle of other viruses that replicate in nucleus such as adenoviruses (Fig. 1g) or retroviruses^46^ deserves further exploration.

The other major player that has been involved in IAV mRNA export so far is the Nuclear Export Factor 1 (NXF1), also known as TAP^18^,^35^. It has been suggested that the intronless, unspliced, and spliced viral mRNAs may harbor differential dependence on NXF1 and/or use distinct adaptors to recruit NXF1^18^,^47^. The three types of viral mRNAs were included in our study. They were all found to depend on DDX19 for their nuclear export (Fig. 4a) and to associate specifically with DDX19 in infected cells (Fig. 5c). Based on the available structural data, DDX19 seems to bind RNA in a non sequence-specific manner, as most contacts are made with the sugar-phosphate backbone^28^,^34^. It is unknown whether small RNAs such as microRNAs and tRNAs, or mRNPs transported by CRM1 rely on DDX19 for their nuclear export^23^,^48^. It is also unclear whether all mRNPs transported by NXF1 undergo DDX19-mediated remodeling, or whether DDX19 is directed to specific mRNPs by adaptor proteins. To our knowledge the only documented case of such an adaptor is the SLIP1 protein, which binds DDX19 and could specifically target it to histone mRNAs^49^. Here, using iPCA (Fig. 5e and ref. 29) and co-immunoprecipitation assays (Fig. 5a), we demonstrate a physical interaction between the viral polymerase and DDX19 in the context of infected cells. We show that this interaction is RNA-independent (Fig. 5a). However, viral mRNAs can be co-purified with the polymerase-DDX19 complex (Fig. 5a,b), suggesting that DDX19 is recruited to viral mRNAs through its interaction with the viral polymerase. Interestingly, the DDX19-polymerase interaction signal was higher in the presence of a E243Q DDX19 mutant able to bind RNA but deficient for ATPase activity^20^, which presumably compromises its ability to remodel exported mRNPs by dissociating RNA-binding protein components from the mRNPs^50^,^51^ (Fig. 5e). This observation suggests that the polymerase-DDX19-mRNA complex is transient and that the viral polymerase dissociates upon ATP hydrolysis-driven remodeling of viral mRNPs, in line with a previous report that viral mRNAs do not stably associate with the viral polymerase in infected cells^52^.

The models that have been proposed for coupling of DDX19 ATPase cycle and DDX19-mediated export of cellular mRNPs are mostly based on *in vitro* studies, which may not accurately reproduce the complexity of the mRNP substrates and the spatial organization of the NPC^3^. One model proposes that the GLE1 nucleoporin ensures loading of DDX19-ATP onto the mRNPs at the cytoplasmic face of the NPC, where ATP hydrolysis-driven remodeling of mRNPs occurs^27^. A second model proposes that mRNPs are initially bound to DDX19-ATP and remodeled upon ATP hydrolysis, after which GLE1 stimulates the release of mRNPs from DDX19-ADP^28^. Our *in cellulo* observations are clearly in favor of the second model, which is also supported by the existence of an intranuclear pool of DDX19 that can associate with mRNPs and with the cellular transcription machinery^22^,^53^. The binding of IAV polymerase to the C-terminal domain of cellular RNA polymerase II early in infection^54^ might facilitate its association with DDX19 and thus the loading of DDX19 (possibly co-transcriptionally) onto viral mRNPs.

Overall, our study identifies a set of diverse (intronless, unspliced and spliced) target mRNAs for DDX19 and sheds light on how DDX19 is directed to these mRNAs. Finally, it points to IAV infection as a very useful model to further elucidate the mechanism and dynamics of DDX19-mediated mRNP remodeling and nuclear export in human cells.

## Methods

### Cells, drugs and viruses

HEK-293T and A549 cells were grown in Dulbecco’s modified Eagle’s medium (DMEM) supplemented with 10% fetal calf serum (FCS). MDCK cells were grown in Modified Eagle’s Medium (MEM) supplemented with 5% FCS. Cycloheximide (CHX, Sigma) was added to the medium at the time of infection (100 μg/mL).

Influenza viruses A/Paris/908/97(H3N2) and A/Udorn/307/72(H3N2) were provided by the National Influenza Center at the Institut Pasteur (Paris, France). The A/WSN/33(H1N1) and A/Paris/650/2004(H1N1) viruses were produced by reverse genetics as described in ref. 55. The VSV (Indiana strain) virus was kindly provided by O. Delmas (Institut Pasteur, Paris, France). The Adenovirus 5 was kindly provided by P. Fortes (Universidad de Navarra, Pamplona, Spain). The recombinant A/WSN/33 viruses expressing a PB2 protein fused to Gluc1 or to the Strep-tag were described previously^29^,^56^. The recombinant A/WSN/33 virus expressing a Nanoluc protein was produced by reverse genetics using a pPolI-PB2-Nanoluc plasmid (described below).

### Plasmids

The Gateway entry plasmids containing the DDX ORFs were obtained from the human ORFeome resource. To generate vectors encoding Gluc2-, GlucFL-, and Strep-DDX fusion proteins, the ORFs were transferred respectively into a Gateway-compatible pSPICA-N2^57^, pGlucFL or pIBA105-GW destination plasmid (kindly provided by C. Demeret and Y. Jacob, respectively). Directed mutagenesis of the Gateway DDX19B entry plasmid was performed using the QuikChange II Site-Directed Mutagenesis Kit (Agilent). The siRNA resistant DDX19B cDNA was designed *in silico* and produced by GenScript.

The reverse genetics plasmid pPolI-PB2-Nanoluc was produced by replacing the Gluc1 sequence of pPolI-PB2-Gluc1 plasmid^29^ with a sequence encoding the self-cleaving 2A peptide from porcine teschovirus followed by the Nanoluc coding sequence, as described in ref. 58. The Nanoluc template was kindly provided by P. Palese and N. Heaton (Icahn School of Medicine at Mount Sinai, New York, USA).

All constructs were verified by Sanger sequencing. The sequences of the oligonucleotides used for amplification and sequencing can be provided upon request.

### Antibodies and immunoblots

Unless otherwise noted, protein extracts were prepared in Laemmli buffer. Immunoblot membranes were incubated with primary antibodies directed against DDX19 (NB100-760, Novus Biologicals), A/PR/8/34 virions^59^, NS1 (kindly provided by Daniel Marc, INRA-Tours, France), M1 (GA2B, Pierce), HA (Genetex), NA (Genetex), GAPDH (Pierce), TPB (Cell Signaling), MEK1/2 (L38C12, Cell Signaling), *Gaussia* luciferase (New England Biolabs), and revealed with secondary antibodies (GE Healthcare) or peroxidase-conjugated Strep-Tactin (IBA), and with the ECL 2 substrate (Pierce). The chemiluminescence signals were acquired using G-Box and the GeneSnap software (SynGene).

### siRNA-based assays

Small interfering RNAs (siRNAs) were purchased from Dharmacon (ON-TARGETplus SMARTpools or individual siRNAs and Non-targeting Control pool). Cells were transfected with 25 nM of siRNA using the DharmaFECT1 transfection reagent (Dharmacon). Cell viability was determined using the CellTiter-Glo Luminescent Viability Assay kit (Promega).

To control the efficiency of siRNAs targeting DDXn genes, siRNA-treated A549 cells were transfected with plasmids encoding a DDXn protein fused with the full-length *Gaussia* luciferase (pGlucFL-DDXn) using polyethylenimine PEI (Polysciences Inc). The luciferase activity was measured 24 h later in cell lysates using the *Renilla* luciferase assay reagent (Promega) and a Berthold CentroXS luminometer.

For screening purpose, A549 cells were infected at 48 hpt with the WSN-PB2-Nanoluc virus at a m.o.i. of 0.0001 pfu/cell. For rescue experiments, HEK-293T cells were transfected with 50 nM of a combination of DDX19A and DDX19B siRNAs using the INTERFERin reagent (Polyplus), and with the pStrep-DDX19B or empty vector using the FuGENE reagent (Promega). The cells were infected at 24 hpt with the WSN-PB2-Nanoluc virus at a m.o.i. of 0.01 pfu/cell, and the luciferase activity was measured at 24 hpi using the Nanoluc Luciferase kit (Promega).

For multi-cycle and single-cycle infection assays, A549 cells were infected at 48 hpt at low and high m.o.i., respectively, as described in ref. 60. Plaque assays on MDCK cells were performed as described in ref. 61. AdV was titrated by immunostaining as described in ref. 62.

### Subcellular fractionation

A549 cells were pelleted at 500 g for 5 min at 4 °C and washed three times with cold PBS. Cell pellets were resuspended in lysis buffer (20 mM HEPES pH 7.9, 3 mM MgCl_2_, 20 mM KCl, 1 mM DTT, 1% Igepal), and incubated for 2 min on ice. Nuclei were pelleted by centrifugation at 500 g for 8 min, and the supernatant was saved as the cytoplasmic fraction. Nuclear pellets were resuspended in low salt buffer (20 mM HEPES pH 7.9, 1.5 mM MgCl_2_, 20 mM KCl, 0.5 mM DTT, 0.2 mM EDTA, 25% glycerol), and high salt buffer (20 mM HEPES pH 7.9, 1.5 mM MgCl_2_, 1.4 M KCl, 0.5 mM DTT, 0.2 mM EDTA, 25% glycerol) was added before incubation on a spinning wheel for 30 min. The insoluble nuclear fraction was pelleted at 10,000 g for 15 min, and the supernatant was saved as the nuclear fraction. Cytoplasmic and nuclear RNAs were extracted using sequentially Trizol LS (Invitrogen) and the RNeasy mini kit (Qiagen).

### RT-qPCR assays

Unless otherwise noted, RNAs were extracted using the RNeasy mini kit (Qiagen). Strand-specific RT-qPCR for NP and NA vRNAs and mRNAs were performed as described in ref. 31. Alternatively, poly(A)+ RNAs were isolated using the Dynabeads mRNA purification kit (Life Technologies). Reverse transcription was performed using the Maxima first strand cDNA synthesis kit (Thermo Scientific), followed by qPCR using SYBR green reagent (Roche) or Solaris qPCR expression assays reagents (Life technologies), and Light Cycler 480 (Roche). For GAPDH and DDX19 transcripts, primers and probes were provided by Thermo Scientific. Primers used for PB2, NP, NA, NS1 and NS2 transcripts were described previously^60^,^63^,^64^. Plasmids containing the sequences of interest were used for calibration curves and absolute quantification. Primers for GAPDH pre-mRNA can be provided upon request.

### Immunoprecipitations and Strep-tag-mediated purifications

Infected HEK-293T were resuspended in lysis buffer (20 mM MOPS-KOH pH 7.4, 120 mM KCl, 0.5% Igepal, 1X Protease Inhibitor Cocktail (Roche) supplemented with 200 U/mL RNasin (Promega) or 100 μg/mL RNase (Thermo Scientific). Clarified lysates were incubated with anti-DDX19 IgG or control IgG for 3 hours. Immuno-complexes were purified with protein A coupled beads (Thermo Scientific). After 3 washes with lysis buffer, elution was performed using Laemmli buffer for protein analyses or RLT buffer (RNeasy mini kit, QIAGEN) for RNA analyses. Strep-tagged proteins of interest were purified using Strep-Tactin beads as described in ref. 65.

### Protein complementation assays

For the infectious protein complementation assay, HEK-293T cells were transfected with the pGluc2-DDX19 plasmids, and subsequently infected with a recombinant WSN virus expressing a PB2 polymerase subunit fused to Gluc1 as described in ref. 29. Normalized Luminescence Ratios (NLRs) were determined as described earlier^29^,^57^.

## Additional Information

**How to cite this article**: Diot, C. *et al*. Influenza A Virus Polymerase Recruits the RNA Helicase DDX19 to Promote the Nuclear Export of Viral mRNAs. *Sci. Rep.* **6**, 33763; doi: 10.1038/srep33763 (2016).

## Supplementary Material

Supplementary Information
