# Supplementary material for: Influenza A Virus Polymerase Recruits the RNA Helicase DDX19 to Promote the Nuclear Export of Viral mRNAs
Source: Sci Rep. 2016 Sep 22;6:33763. doi: 10.1038/srep33763 (PMC5037575; doi:10.1038/srep33763)
Supplement: Supplementary Information [file srep33763-s1.pdf]

Influenza A Virus Polymerase Recruits the RNA Helicase DDX19 to Promote the Nuclear Export of Viral mRNAs

Cédric Diot[1,2,3], Guillaume Fournier[1,2,3], Mélanie Dos Santos[1,2,3], Julie Magnus[1,2,3], Anastasia Komarova[2,4], Sylvie van der Werf[1,2,3], Sandie Munier[1,2,3¶], Nadia Naffakh[1,2,3¶*]

[1] Institut Pasteur, Unité de Génétique Moléculaire des Virus à ARN, Département de Virologie, F-75015 Paris, France

[2] CNRS, UMR3569, F-75015 Paris, France

[3] Université Paris Diderot, Sorbonne Paris Cité, Unité de Génétique Moléculaire des Virus à ARN, EA302, F-75015 Paris, France

[4] Institut Pasteur, Unité de Génomique Virale et Vaccination, Département de Virologie, F-75015 Paris, France

[¶]co-senior authors

*corresponding author: nadia.naffakh@pasteur.fr

Table S1. List of human DDX proteins.

Gene Symbol	Synonyms	NCBI Gene ID	NCBI RefSeq protein	Included in the interaction screen
**Bona-fide DDX proteins**				
**DDX2A**	EIF4A1	1973	NP_001407.1	✔
**DDX2B**	BM-010, eIF4B, EIF4A2	1974	NP_001958.2	✔
**DDX3X**	DDX14, HLP2	1654	NP_001180345.1	✔
**DDX3Y**		8653	NP_004651.2	✗
**DDX4**	VASA	54514	NP_001160005.1	✗
**DDX5**	p68	1655	NP_004387.1	✔
**DDX6**	p54, RCK, HLR2	1656	NP_004388.2	✔
**DDX10**		1662	NP_004389.2	✔
**DDX17**	p72	10521	NP_006377.2	✔
**DDX18**	MrDb	8886	NP_006764.3	✔
**DDX19A**	Dbp5, Rat8	55308	NP_060802.1	✔
**DDX19B**		11269	NP_009173.1	✔
**DDX20**	DP103, Gemin3	11218	NP_009135.4	✔
**DDX21**	GURDB, RH-II/GU	9188	NP_004719.2	✔
**DDX23**	U5-100K	9416	NP_004809.2	✔
**DDX24**		57062	NP_065147.1	✔
**DDX25**	GRTH	29118	NP_037396.3	✔
**DDX27**	DRS1	55661	NP_060365.7	✔
**DDX28**	MDDX28	55794	NP_060850.2	✔
**DDX31**		64794	NP_073616.6	✔
**DDX39A**	URH49, BAT1	10212	NP_005795.2	✔
**DDX39B**	BAT1, UAP56	7919	NP_004631.1	✗
**DDX41**	ABS	51428	NP_057306.2	✔
**DDX42**	RHELP, SF3b125	11325	NP_031398.2	✔
**DDX43**	HAGE	55510	NP_061135	✗
**DDX46**		9879	NP_055644.2	✔
**DDX47**		51202	NP_057439.2	✔
**DDX48**	eIF4A3, eIF4A-III	9775	NP_055555.1	✔
**DDX49**		54555	NP_061943.2	✔
**DDX50**	Gu-beta, RH-II/GuB	79009	NP_076950.1	✔
**DDX51**		317781	NP_778236.2	✔
**DDX52**	ROK1	11056	NP_008941.3	✔
**DDX53**	CAGE	168400	NP_874358.2	✗
**DDX54**	DP97	79039	NP_001104792.1	✔
**DDX55**		57696	NP_065987.1	✔
**DDX56**	NOH61	54606	NP_061955.1	✔
**DDX59**	ZNHIT5	83479	NP_001026895.2	✔
**Putative DDX proteins**				
**DDX1**	DBP-RB	1653	NP_004930.1	✔
**DDX11**	CHLR1, KRG2	1663	NP_689651.1	✔
**DDX12**	CHLR2	440081	n.a.	✗
**DDX13**	SKI2W, SKIV2	6499	NP_008860.4	✔
**DDX58**	RIG-I	23586	NP_055129	✗
**DDX60**	DDX60-L	55601	NP_060101	✗

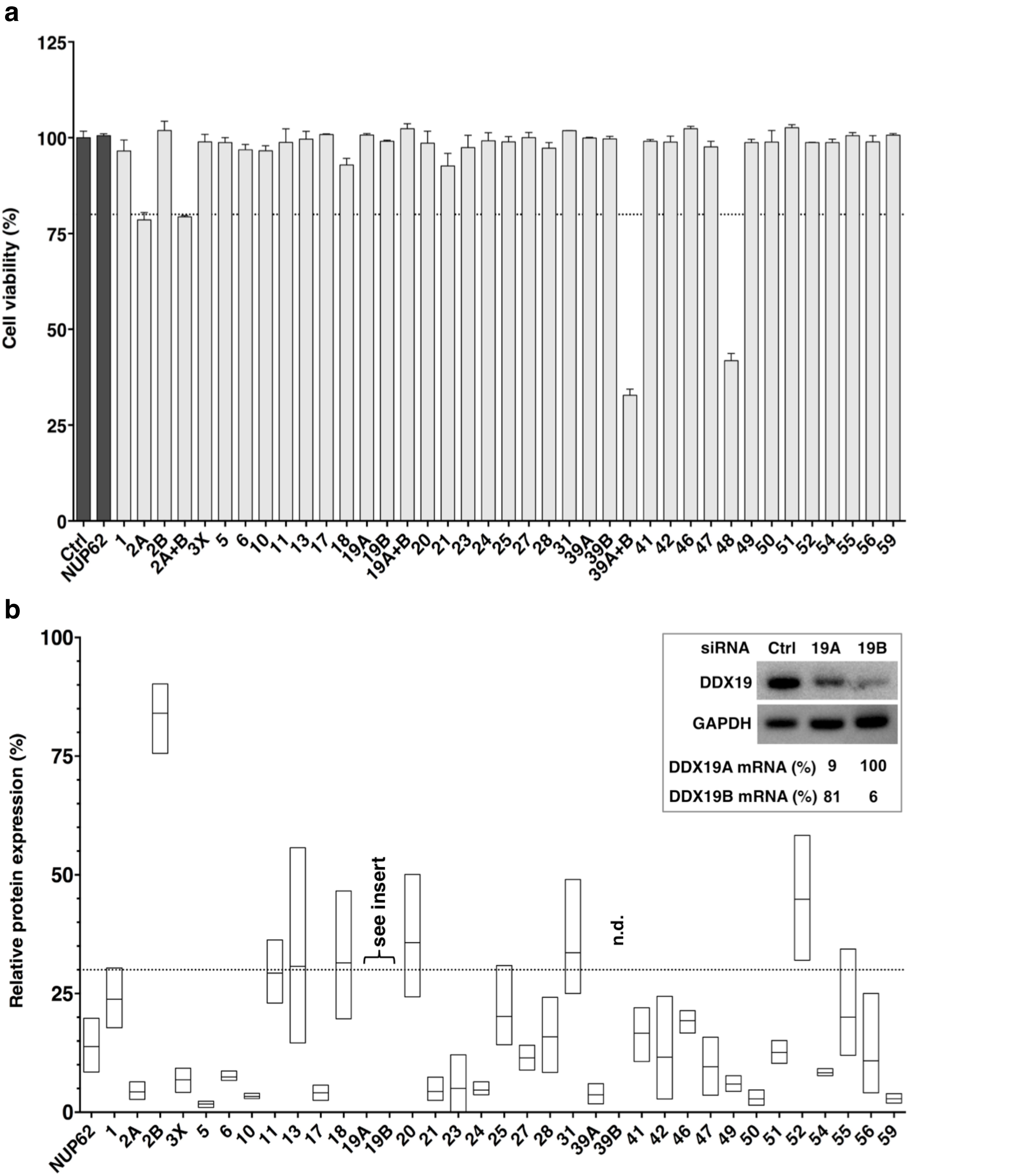
a
Cell viability (%)
125
100
75
50
25
0
Ctrl
NUP62
1
2A
2B
2A+B
3X
5
6
10
11
13
17
18
19A
19B
19A+B
20
21
23
24
25
27
28
31
39A
39B
39A+B
41
42
46
47
48
49
50
51
52
54
55
56
59
b
Relative protein expression (%)
100
75
50
25
0
see insert
n.d.
siRNA
Ctrl
19A
19B
DDX19
GAPDH
DDX19A mRNA (%) 9 100
DDX19B mRNA (%) 81 6
NUP62
1
2A
2B
3X
5
6
10
11
13
17
18
19A
19B
20
21
23
24
25
27
28
31
39A
39B
41
42
46
47
49
50
51
52
54
55
56
59

Figure S1

Figure S1. Toxicity and silencing efficiency of siRNAs.

(a) A549 cells were transfected with 25 nM of siRNA and cell viability was determined at 72 hours post-transfection (hpt) using the CellTiter-Glo Luminescent Viability Assay kit (Promega). The results are expressed as the mean percentages ± SEM. When mean signals were below 80% (dashed line), siRNAs were considered as being toxic.

(b) A549 cells were transfected with 25 nM of control or DDXn-targeting siRNAs and with plasmids encoding the corresponding DDXn protein fused with the full-length *Gaussia* luciferase (pGlucFL-DDXn). Ratios of the luciferase activities obtained in cells transfected with the DDX-targeting siRNA to the one obtained in cells transfected with the control siRNA are shown. The results are represented as floating bars with a line at the mean. The dashed line corresponds to a relative protein expression of 30% in silenced cells compared to control cells. n.d.: not determined.

Insert: At 48 hpt, the levels of DDX19 protein were evaluated by immunoblot using an antibody that recognizes both A and B forms of DDX19, and the levels of DDX19A and DDX19B mRNAs were determined by RT-qPCR and expressed as percentages of the control. Cropped blots are shown. The corresponding full-length blots are shown in Figure S10.

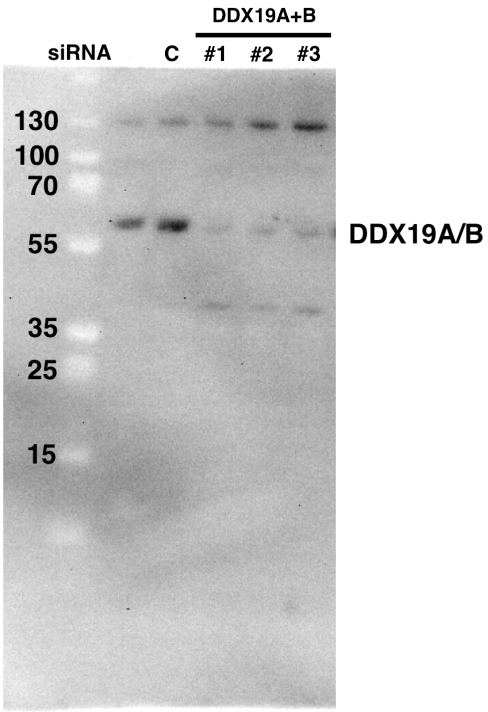

Figure S2a

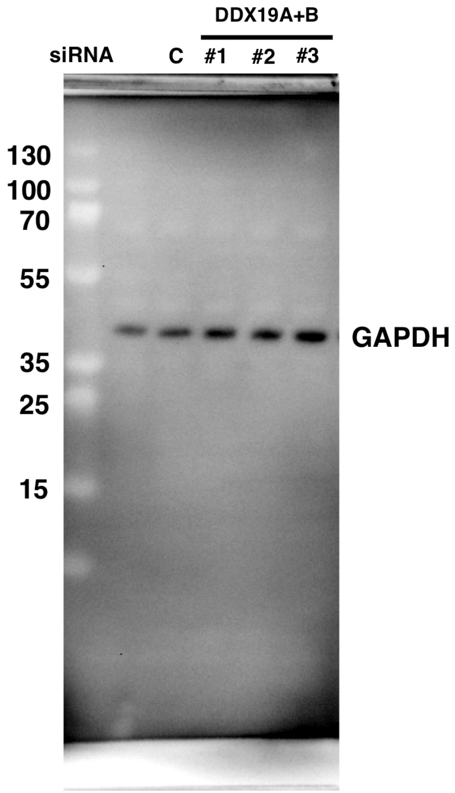

Figure S2b

Figure S2 related to Figure 1c.

A549 cells were treated with control non-target siRNAs (C) or individual siRNAs targeting DDX19A+B (#1, #2 and #3). (a) At 48 hours post-transfection, the levels of DDX19 protein were evaluated by immunoblot using an antibody that recognizes both A and B forms of DDX19. (b) The membrane was rehybridized with an anti-GAPDH antibody.

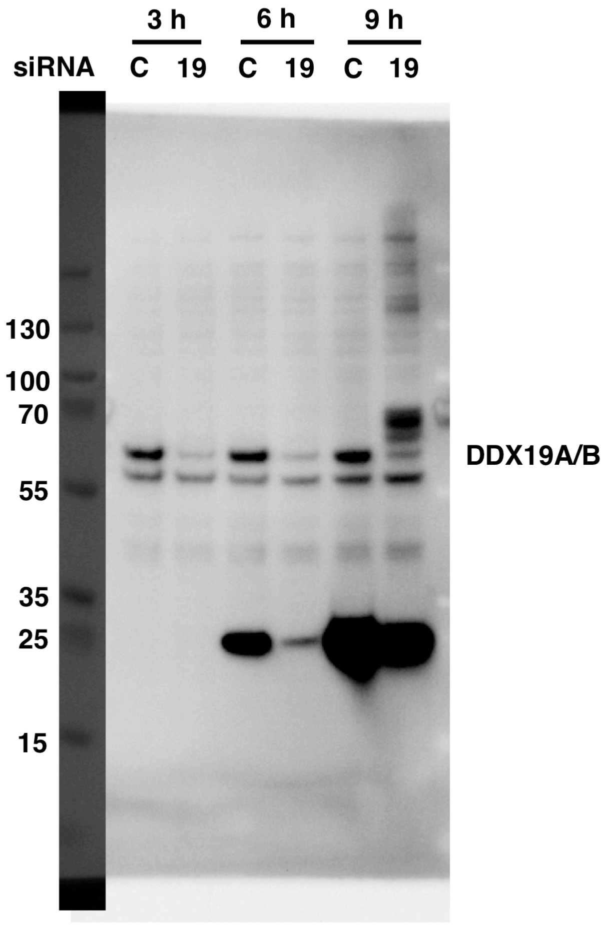

Figure S3a

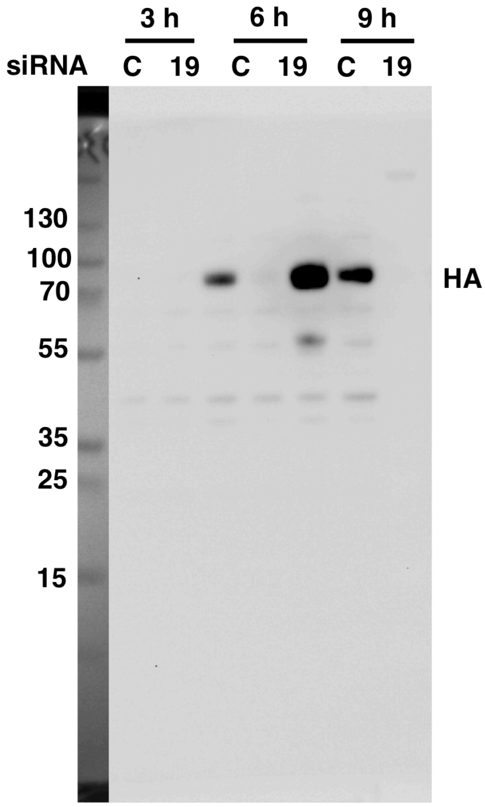

Figure S3b

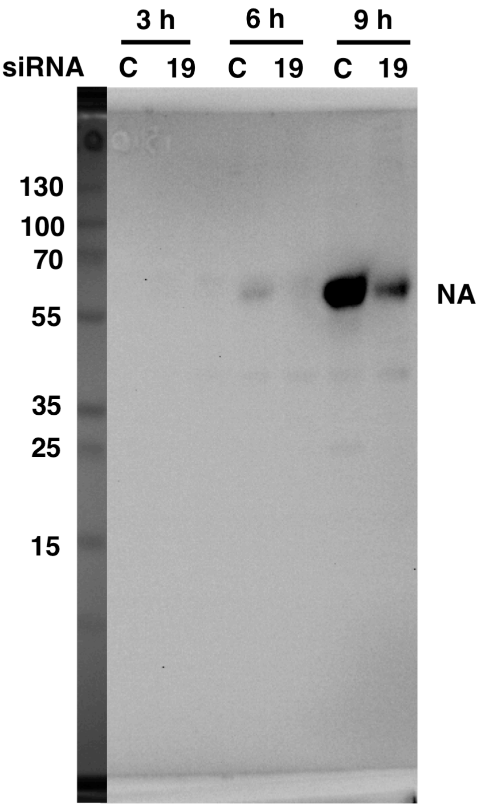

Figure S3c

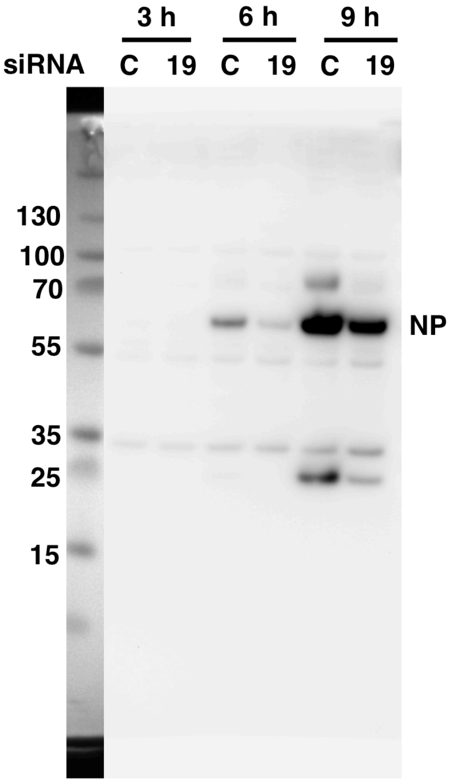

Figure S3d

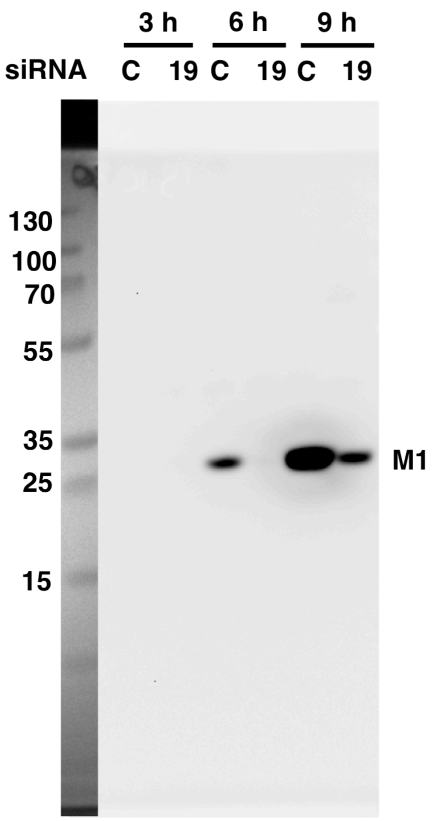

Figure S3e

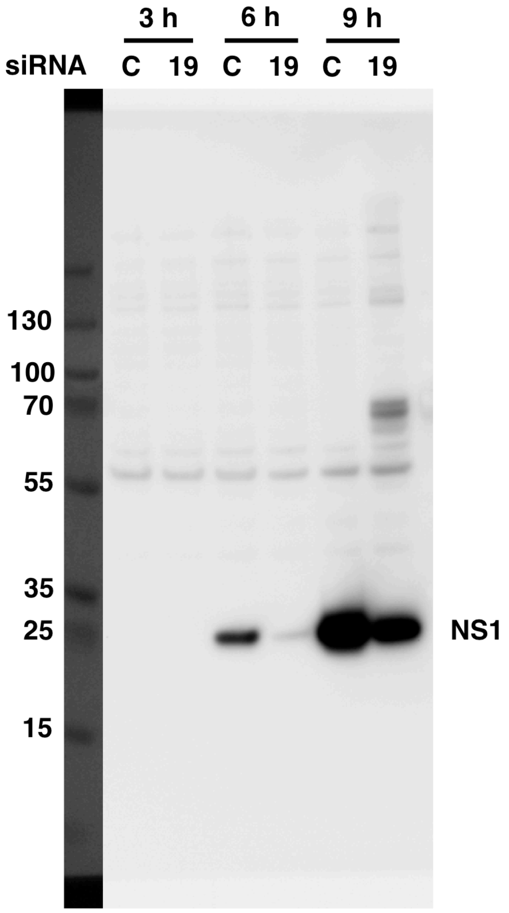

Figure S3f

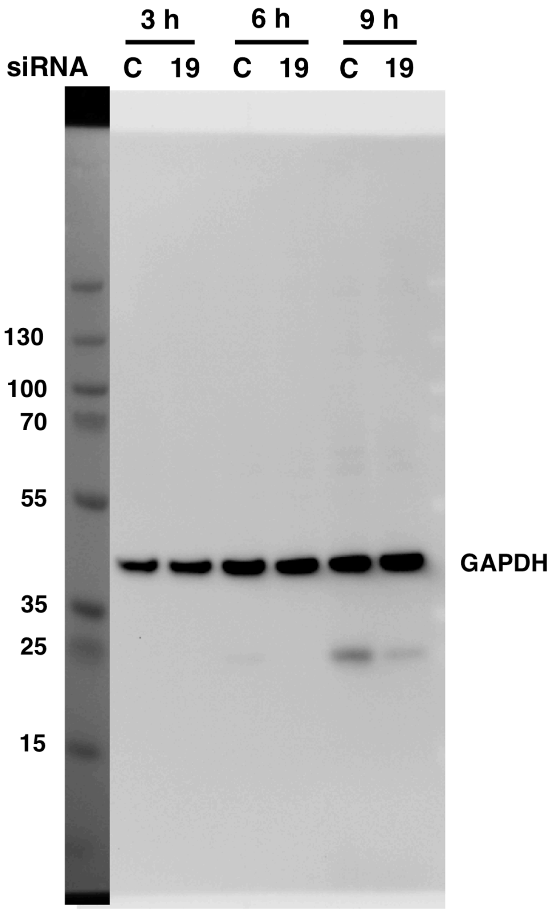

Figure S3g

Figure S3 related to Figure 2a.

A549 cells were treated with control (C) or DDX19 (19) siRNAs and infected with WSN (5 pfu/cell). Total extracts were prepared at the indicated times post-infection and analyzed by immunoblots using antibodies directed against DDX19A/B (a), HA (b), NA (c), NP (d), M1 (e), NS1 (f), or GAPDH (g). C: control siRNAs; 19: DDX19 siRNAs. Signals in (a), (f) and (g) result from serial hybridizations of the same membrane with different antibodies. Residual signals resulting from the initial hybridization with the anti-NS1 antibody (f) is visible in (a) and (g).

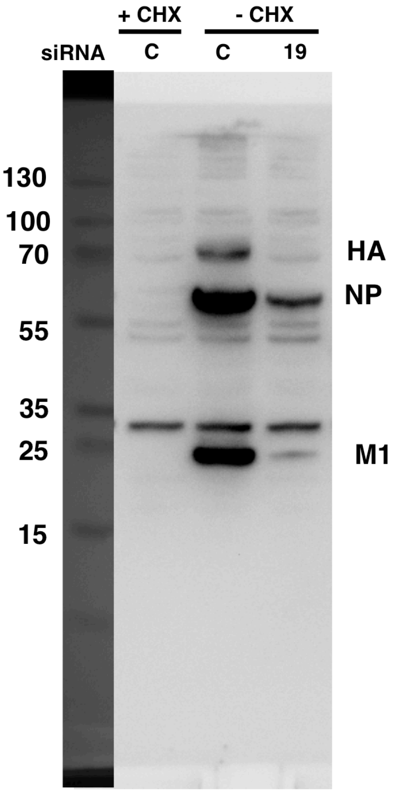

Figure S4a

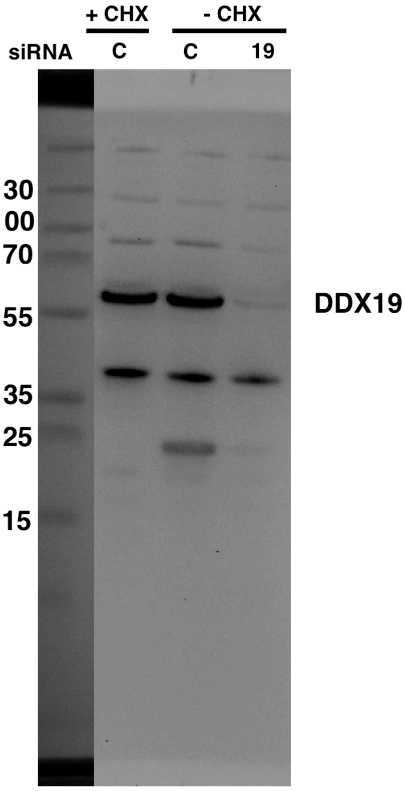

Figure S4b

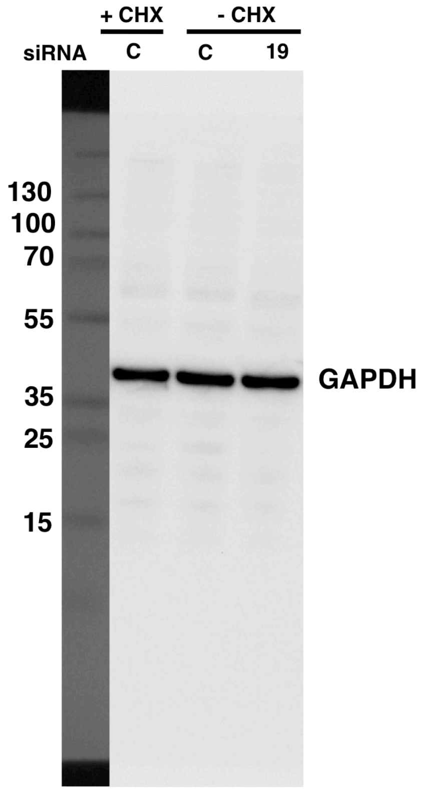

Figure S4c

Figure S4 related to Figure 3a.

A549 cells treated with control or DDX19 siRNAs were infected with WSN (5 pfu/cell) in the presence of cycloheximide (CHX; 100 µg/mL). Total extracts from control cells treated with CHX (+ CHX) or not (- CHX) were prepared at 6 hpi and analyzed by immunoblots using antibodies directed against the A/PR/8/34 virions (allowing the simultaneous detection of HA, NP and M1) (a), DDX19A/B (b) or GAPDH (c). C: control siRNAs; 19: DDX19 siRNAs. Signals in (b) and (c) result from serial hybridizations of the same membrane with different antibodies.

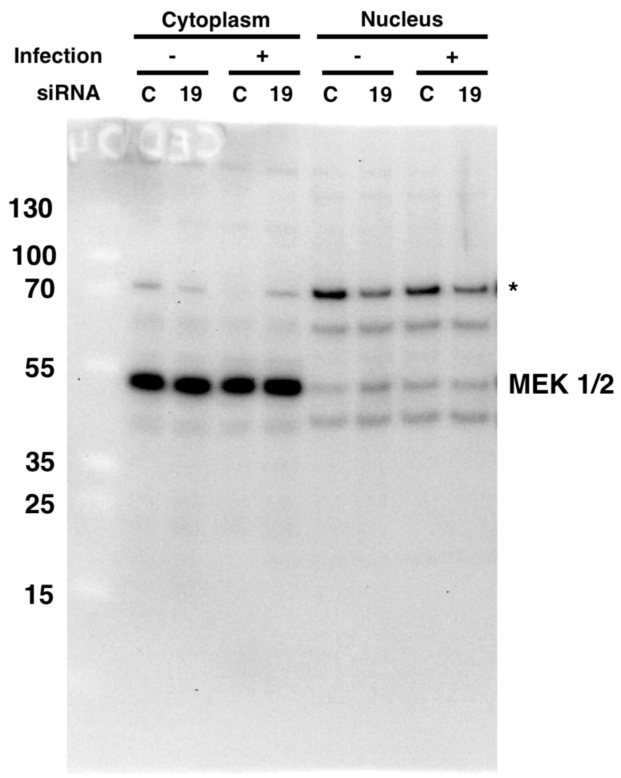

Figure S5a

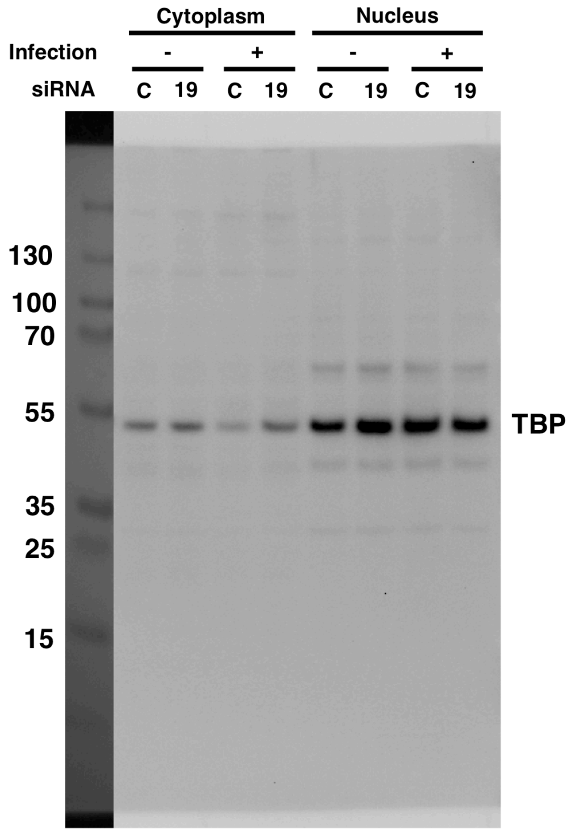

Figure S5b

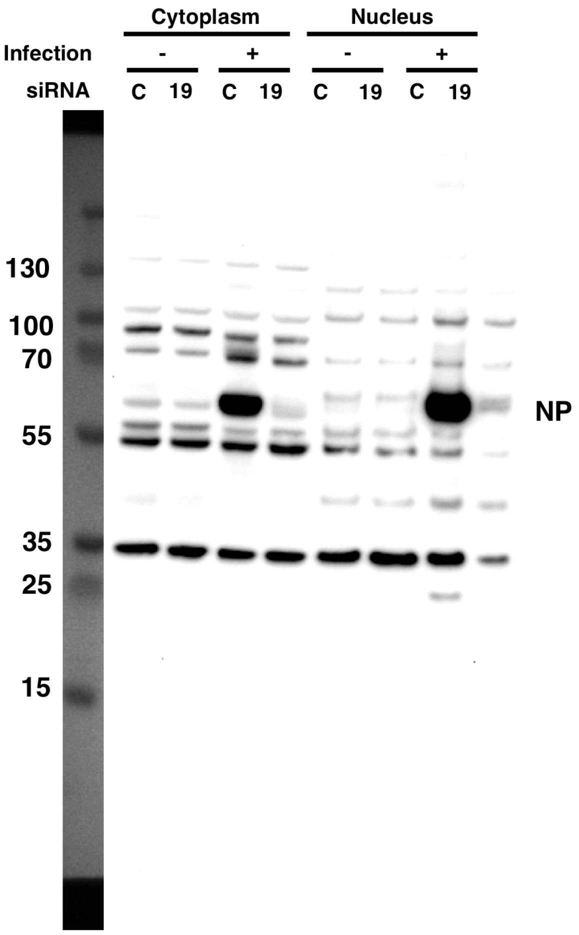
Cytoplasm
Nucleus
Infection
-
+
-
+
siRNA
C
19
C
19
C
19
C
19
130
100
70
55
35
25
15
NP

Figure S5c

Figure S5 related to Figure 3b.

Cytoplasmic and nuclear fractions from A549 cells treated with control or DDX19 siRNAs, infected or not with WSN (5 pfu/cell), were prepared. Immunoblots analysis was performed using antibodies directed against MEK1/2 (a), TBP (b) or NP (c). C: control siRNAs; 19: DDX19 siRNAs. In (a), the band indicated by a star corresponds to the lamin, as revealed upon hybridization with an anti-lamin antibody previous to the hybridization with an anti-MEK1/2 antibody.

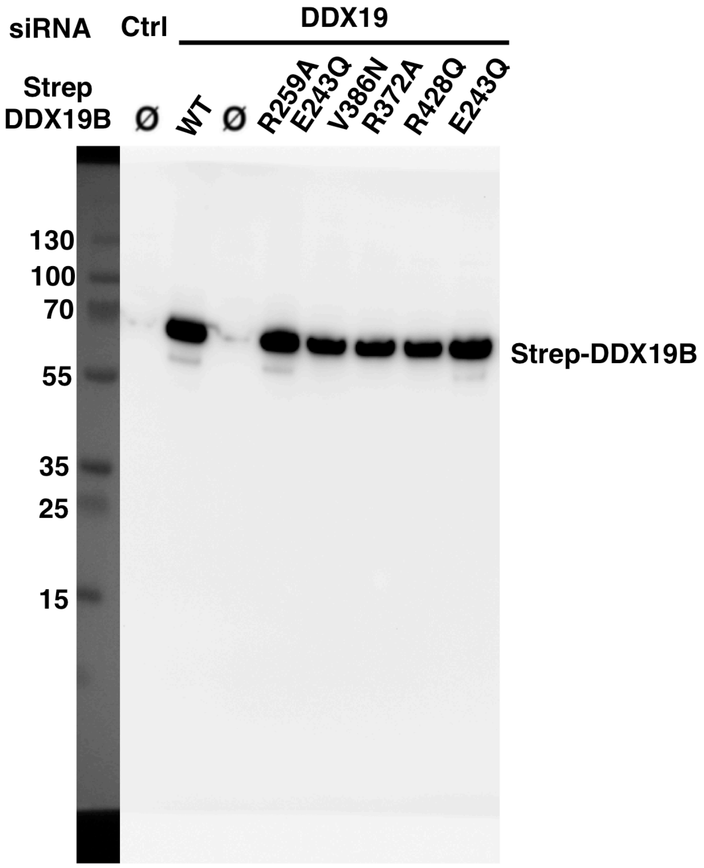

Figure S6a

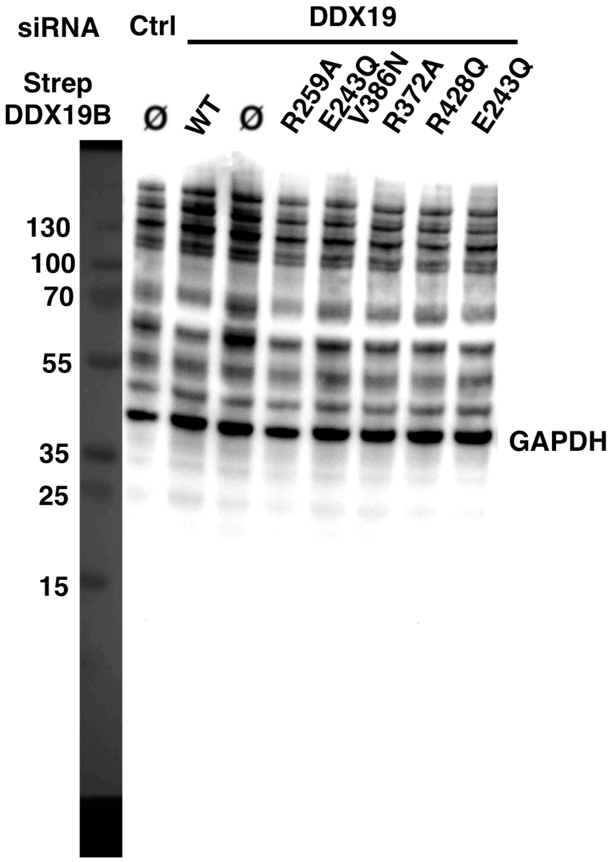

Figure S6b

Figure S6 related to Figure 4d.

HEK-293T cells were transfected with DDX19 or control siRNAs and then with siRNA-resistant DDX19B plasmids or empty vector. After 24 h, cells were infected with the WSN-PB2-Nanoluc virus (0.01 pfu/cell). (a) Lysates from the RNAi rescue experiment shown in Figure 4c were analyzed by immunoblot using Strep-Tactin. (b) The membrane was rehybridized with an anti-GAPDH antibody. The ⦸ symbol indicates cells transfected with the empty vector.

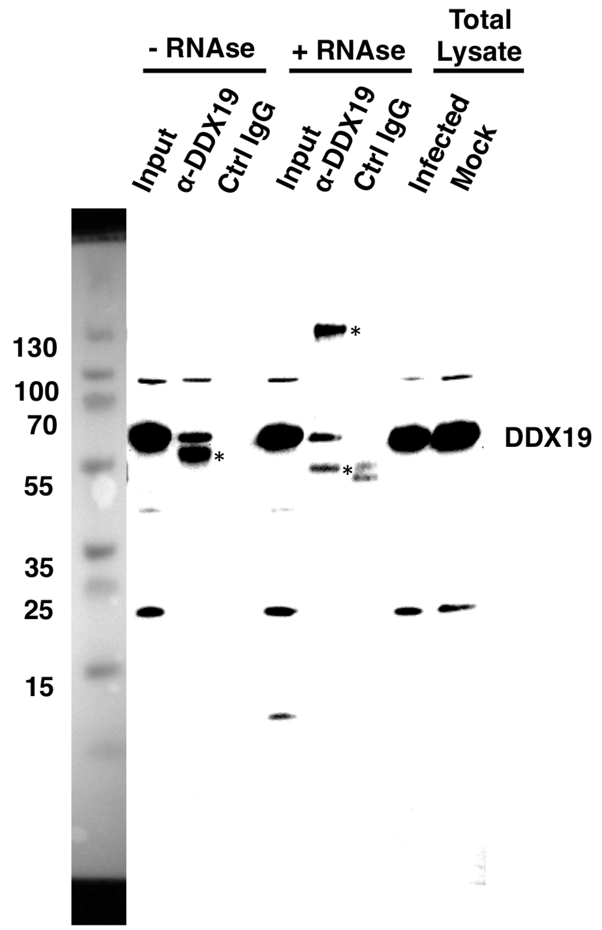

Figure S7a

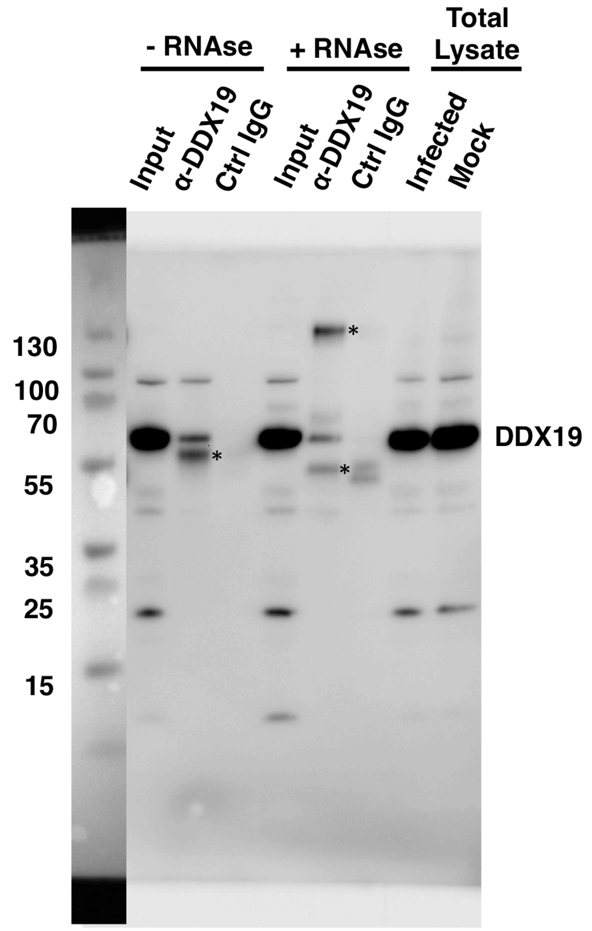

Figure S7b

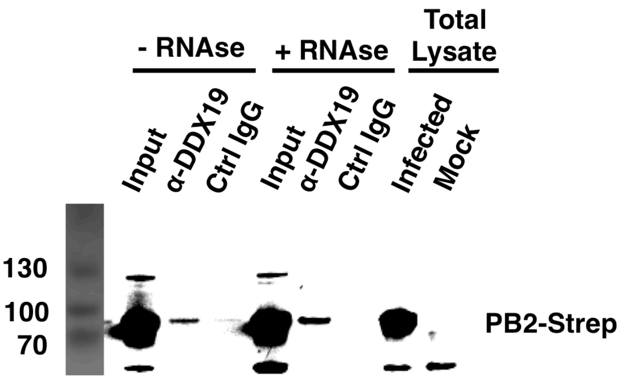

Figure S7c

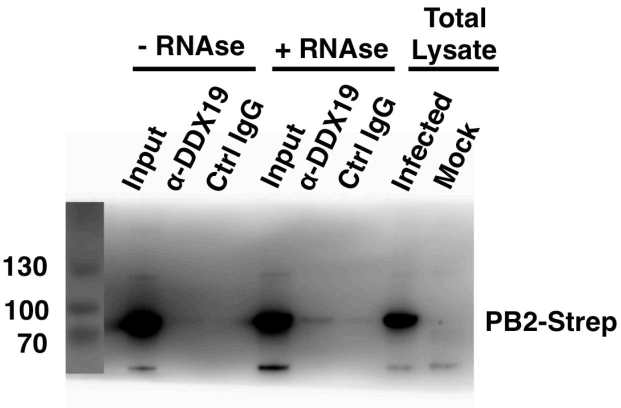

Figure S7d

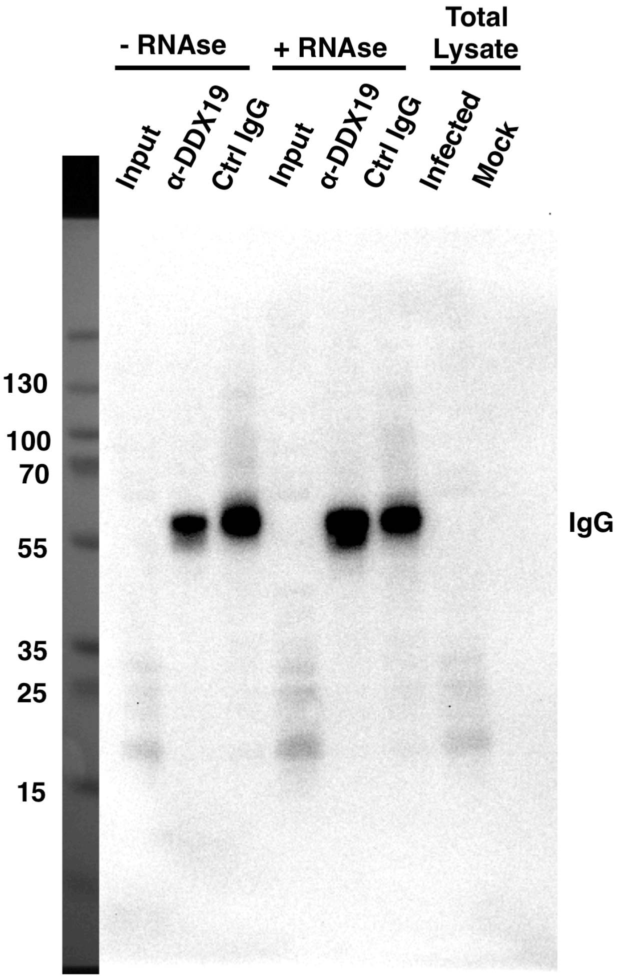

Figure S7e

Figure S7 related to Figure 5a.

A549 cells were infected with WSN (5 pfu/cell). At 3.5 hpi, DDX19 proteins were purified in the absence or presence of RNAse, using anti-DDX19 antibodies (α-DDX19) or control immunoglobulins (Ctrl IgG). Inputs and α-DDX19 / Ctrl IgG eluates were analyzed by immunoblot using anti-DDX19 antibodies (a), Strep-Tactin (c), or anti-rabbit IgG antibodies (e). (b) and (d) are identical to (a) and (b), respectively, but at a lower exposure. (c and d) Due to chemiluminescence detection reagent shortage, the membrane was cut prior to its exposure to the reagent. Results representative of two independent experiments are shown. In (a) and (b), the stars indicate bands that are not formally identified but could correspond to cellular proteins that are non-specifically immunoprecipitated and detected by anti-DDX19 antibodies, or to degradation products of DDX19 that are preferentially immunoprecipitated.

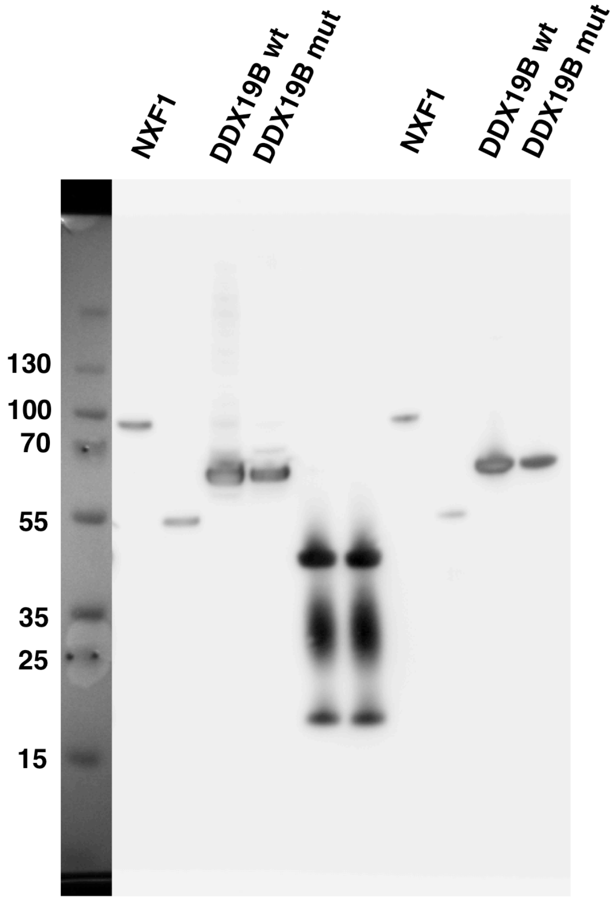

Figure S8a

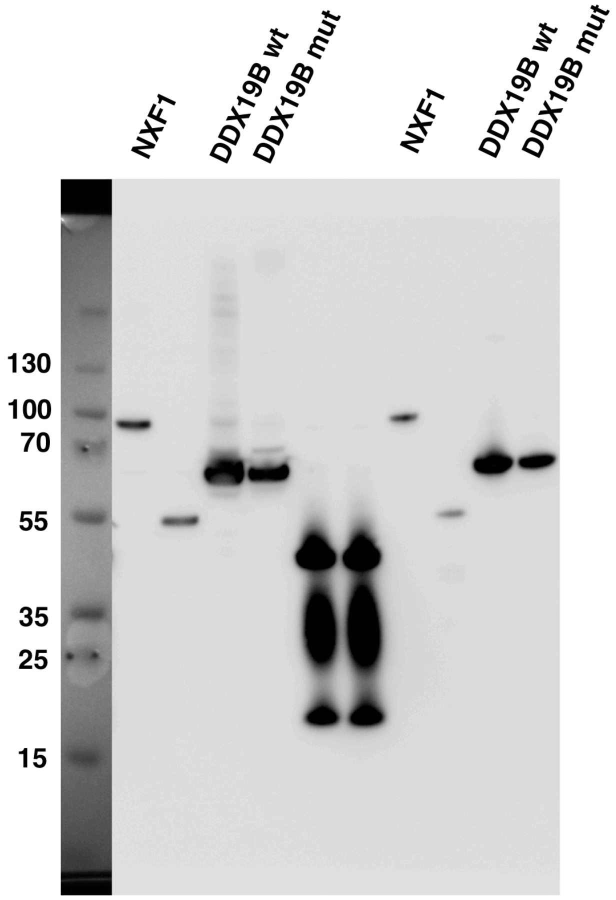

Figure S8b

Figure S8 related to Figure 5d.

(a and b) HEK-293T cells expressing Strep-tagged wild-type DDX19B, mutant DDX19B or NXF1 proteins were infected with WSN (5 pfu/cell). At 6 hpi, Strep-tagged proteins were purified. Lysates and eluates were analyzed by immunoblot using Strep-Tactin. (b) is identical to (a) but at a higher exposure.

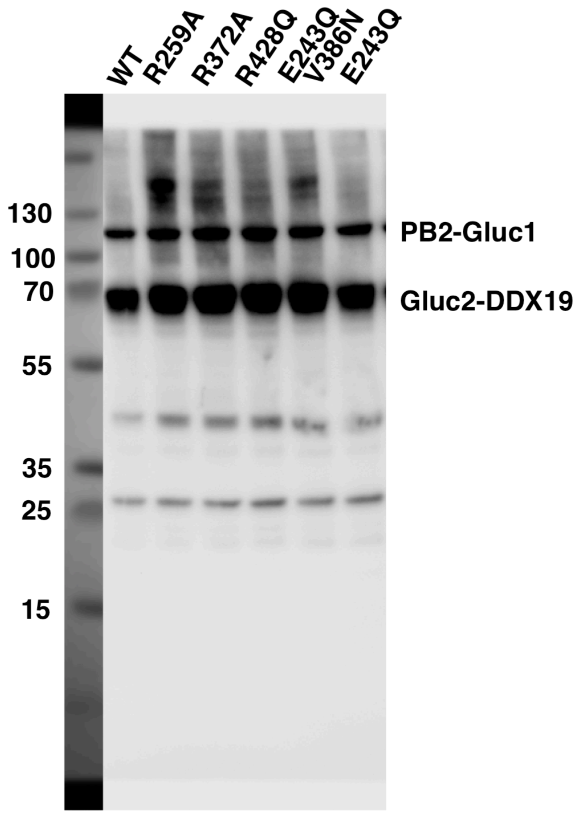

Figure S9a

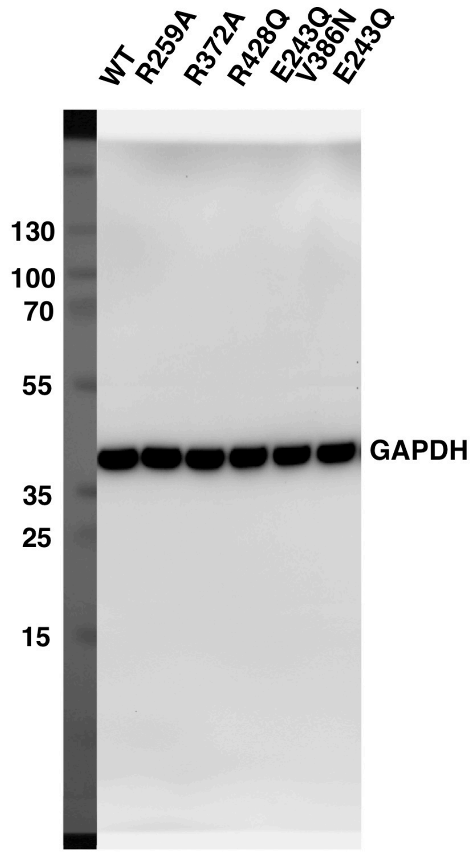

Figure S9b

Figure S9 related to Figure 5f.

HEK-293T cells expressing the Gluc2-DDX19B wild-type or mutant proteins were infected with the WSN-PB2-Gluc1 virus at a m.o.i. > 1 pfu/cell. (a) Cell lysates from the iPCA experiment shown in Figure 5e were analyzed by immunoblot using anti-Gluc. (b) The membrane was rehybridized with an anti-GAPDH antibody.

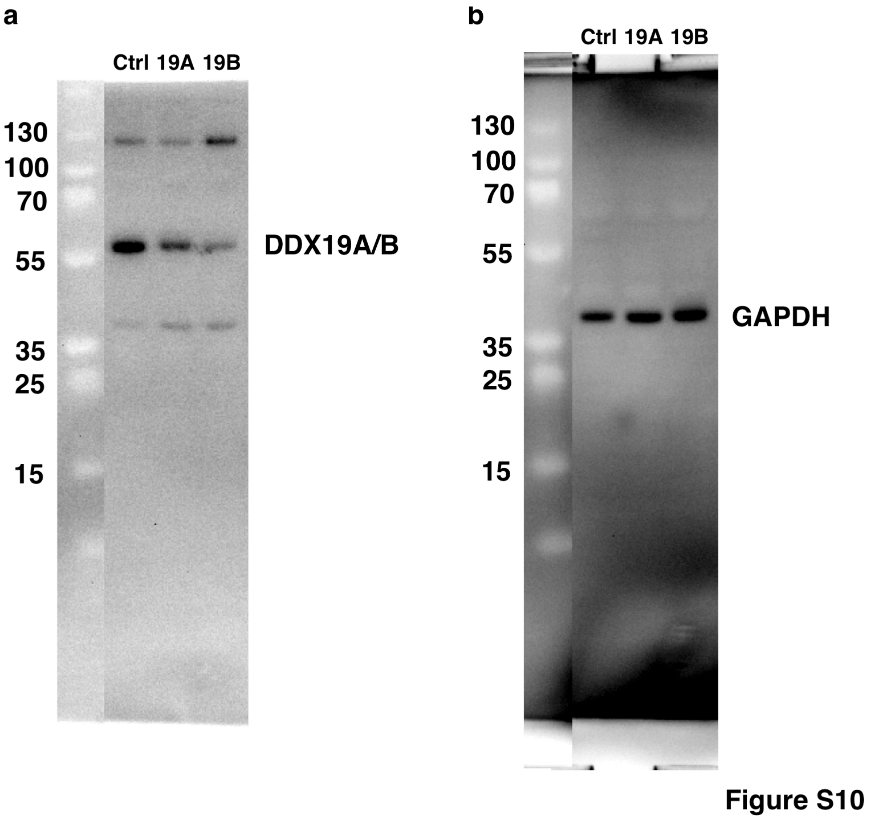

Figure S10 related to Figure S1.

A549 cells were transfected with 25 nM of control, DDX19A- or DDX19B-targeting siRNAs. (a) At 48 hpt, the levels of DDX19 protein were evaluated by immunoblot using an antibody that recognizes both A and B forms of DDX19. (b) The membrane was rehybridized with an anti-GAPDH antibody.
